# Assessment and management of magnesium and trace element status in children with CKD stages 2–5, on dialysis and post-transplantation: Clinical practice points from the Pediatric Renal Nutrition Taskforce

**DOI:** 10.1007/s00467-025-06759-5

**Published:** 2025-05-17

**Authors:** Jetta Tuokkola, Caroline E. Anderson, Sheridan Collins, Pearl Pugh, Molly R. Wong Vega, Matthew Harmer, Lyndsay A. Harshman, Christina L. Nelms, Barry Toole, An Desloovere, Fabio Paglialonga, Nonnie Polderman, José Renken-Terhaerdt, Rukshana Shroff, Evelien Snauwaert, Stella Stabouli, Johan Vande Walle, Bradley A. Warady, Vanessa Shaw, Larry A. Greenbaum

**Affiliations:** 1 Clinical Nutrition Unit, Internal Medicine and Rehabilitation, University of Helsinki and Helsinki University Hospital, Helsinki, Finland; 2 Institute of Public Health and Clinical Nutrition, University of Eastern Finland, Kuopio, Finland; 3 University Hospital Southampton NHS Foundation Trust, University of Southampton, Southampton, UK; 4 Children’s Hospital Westmead, Sydney Children’s Hospital Network, Sydney, Australia; 5 Nottingham Children’s Hospital, Queen’s Medical Centre, Nottingham, UK; 6 Texas Children’s Hospital, Houston, TX USA; 7 Stead Family Department of Pediatrics, University of Iowa, Iowa City, IA USA; 8 Children’S Mercy Kansas City, Kansas City, MO USA; 9 Department of Blood Sciences, Freeman Hospital, Newcastle Upon Tyne, UK; 10 Department of Pediatric Nephrology, Ghent University Hospital, Ghent, Belgium; 11 Fondazione IRCCS Ca’ Granda Ospedale Maggiore Policlinico, Milan, Italy; 12 British Columbia Children’s Hospital, Vancouver, Canada; 13 Wilhemina Children’S Hospital, University Medical Center Utrecht, Utrecht, The Netherlands; 14 University College London Great Ormond Street Hospital Institute of Child Health, London, UK; 15 1st Department of Pediatrics, Aristotle University, Hippokratio Hospital, Thessaloniki, Greece; 16 Emory University and Children’s Healthcare of Atlanta, Atlanta, GA USA

**Keywords:** Magnesium, Minerals, Trace elements, Chronic kidney disease, Pediatric Renal Nutrition Taskforce, Clinical practice points, Children

## Abstract

Children and young people with chronic kidney disease (CKD) are at risk for deficiency or excess of magnesium and trace elements. Kidney function, dialysis, medication, and dietary and supplemental intake can affect their biochemical status. There is much uncertainty about the requirements of magnesium and trace elements in CKD, which leads to variation in practice. The Pediatric Renal Nutrition Taskforce is an international team of pediatric kidney dietitians and pediatric nephrologists, formed to develop evidence-based clinical practice points to improve the nutritional care of children with CKD. PICO (patient, intervention, comparator, and outcomes) questions led the literature searches, which were conducted to ascertain current biochemical status, dietary intake, and factors leading to requirements differing from healthy peers, and to guide nutritional care of children with CKD stages 2–5, on dialysis, and post-transplantation. We address the assessment and intervention of magnesium and the trace elements chromium, copper, fluoride, iodine, manganese, selenium, and zinc. We suggest routine biochemical assessment of magnesium. Trace element assessment is based on clinical suspicion of deficiency or excess and their risk factors, including accumulation, losses, medications, nutrient interactions, and comorbidities. In particular, we suggest assessing magnesium, copper, iodine, and zinc when growth is poor, and evaluating magnesium, copper, selenium, and zinc in the presence of proteinuria. A structured approach to magnesium and trace element management, including biochemical, physical, and dietary assessment, is beneficial in the paucity of evidence. Research recommendations are suggested.

**Supplementary Information:**

The online version contains supplementary material available at 10.1007/s00467-025-06759-5.

## Introduction

Magnesium (Mg) and trace elements (TEs) are required for essential roles in body processes [1], and body stores can be affected by diet, supplements, kidney function, urine loss, exogenous sources, and dialysis. Deficiency and excess are more common in chronic kidney disease (CKD) and may cause adverse clinical consequences, such as poor growth [2]. Previous pediatric guidelines for mineral and TE management specific to children with CKD are over 15 years old [3, 4] and are based on limited evidence with the absence of attention to some key TEs. The principal messages from these guidelines are that dietary intake should provide 100% of the requirements for healthy children and that supplementation should be considered when there is inadequate intake or clinical evidence of deficiency.

We address the mineral Mg and the TEs chromium (Cr), copper (Cu), fluoride (Fl), iodine (“I”), manganese (Mn), selenium (Se), and zinc (Zn). We present a comprehensive position paper with six clinical practice points (CPPs) to address Mg and TE status, intervention, and monitoring for children with CKD stages 2–5, on dialysis and post-transplantation (CKD2–5D/T). This builds upon the 2009 Kidney Disease Outcomes Quality Initiative recommendations [4] using data from more recent literature and provides new guidance for Mg, Cu, Cr, Fl, “I”, Mn, Se, and Zn in children. These CPPs are based on available evidence, expert opinion and, where appropriate, extrapolation from adult studies. Calcium and phosphate [5], potassium [6], sodium, and iron are out of scope of this CPP and have either been addressed in previous documents or will be discussed in subsequent guidelines.

## Methods

A previous Pediatric Renal Nutrition Taskforce (PRNT) publication has described the full development process utilized for consensus recommendations [5]. The search criteria are described in Table S1. The PRNT working group, which included pediatric nephrologists, dietitians, and biochemists, defined the scope, formulated the questions, performed the literature review, developed the CPPs, and conducted the Delphi survey. The Delphi group included nephrologists and dietitians from global pediatric kidney centers.

### Development process

PICO (patient, intervention, comparator, and outcome) questions guided the development of actionable CPPs.

A ***PICO questions***
  *Population/patient*: children from birth up to 18 years of age with CKD.
  *Intervention*: assessment of Mg and TE requirements, intake, and clinical evidence of deficiency or excess; use of supplementation, and monitoring.
  *Comparison*: Mg and TE requirements for healthy children and adults with CKD and biochemical markers, where available (or no comparator).
  *Outcomes:* adequate Mg and TE intake; avoidance of deficiency and excess.
B ***Proposed clinical questions for CPPs for children with CKD***
  1. What are the Mg and TE requirements?
  2. What are the main dietary sources of Mg and TEs?
  3. How do dietary modifications affect intake?
  4. What other factors influence Mg and TE intake and status?
  5. How should we approach clinical assessment and monitoring?
  6. When is dietary modification of Mg or TE supplementation indicated?

### Literature search

Table S1 details the literature search*.* Literature up to and including May 2024 was reviewed using PICO and related clinical questions.

### CPPs

1.0 What are the magnesium and trace element requirements?

1.1 The magnesium and trace element requirements for most children with CKD should approximate those of healthy children of the same chronological age.

1.2 For some children with CKD, magnesium, and trace element requirements may be less or greater than those for healthy children.What are the magnesium and trace element requirements.

#### Evidence and rationale

We first describe the requirements for healthy children as a comparator to understand the requirements for children with CKD. Table S2 summarizes internationally published recommended dietary intakes of Mg and TEs. Table S3 presents the requirements’ definitions.

The requirements of Mg and TEs for children and young people with CKD have not been determined. Therefore, as an initial guide, the requirements for healthy children should be used, with the exception of advanced CKD (stages 3b to 5D) when Mg and certain TEs, namely “I”, begin to accumulate, and others are lost through dialysis (see “What other factors influence magnesium and trace element status?” for details). The exception is Fl, which accumulates in earlier stages of CKD.

In the early stages of CKD, we recommend increasing intake of Mg and TEs via diet or supplementation, if dietary intake is lower than recommended for healthy children. Children on dialysis may benefit from a higher intake of TEs, but this depends on the quantity of TEs removed by dialysis, which again is dependent on dialysate concentration.

2.0 What are the main dietary sources of magnesium and trace elements?

2.1 Children with CKD may achieve sufficient intake of magnesium and trace elements from a varied diet, breast milk, infant formula, or a nutritionally complete enteral formula.

#### Evidence and rationale

Dietary modifications in CKD to manage uremia, hyperkalemia, hyperphosphatemia, and hyperlipidemia may lead to restrictions of dietary sources. Table 1 shows the main dietary sources for Mg and each TE and the impact of dietary modifications. A varied diet can help optimize Mg and TE intake.

3.0 How do dietary modifications affect intake?

3.1 Dietary modifications and food preparation methods may impact magnesium and trace element intake.

**Table 1:** Magnesium and trace elements impacted by potential dietary modifications

	Meat*^†‡ A^	Nuts^†^, seeds, and meat alternatives	Fish*^†‡^	Dairy*^†‡^** and egg*^†^	Fruit^‡^	Vegetables^‡^	Breads and cereals^†‡^	Drinks^‡^	Processed foods/other*^†‡^**
Magnesium		Nuts^†^ (e.g., Brazil nuts), seeds (e.g., sunflower, pumpkin, and chia), peanut butter	Fish*^†‡^ and seafood*^†‡^	Yoghurt*^†‡^**, milk*^†‡^**, and cheese*^†‡^**	Berries, bananas^‡^	Green leafy vegetables (e.g.. spinach^‡^ and legumes), avocados^‡^, artichoke	Whole grains, grain products (possibly ^†‡^) (e.g,. breakfast cereals, wholegrain and seeded breads, brown rice, quinoa, and edamame	Coffee^‡^ and cocoa beverages Bottled/tap water, “hard” water, and mineral water^‡^ Soy beverages^‡^ and fruit juices^‡^	Dark chocolate^†‡^ Low sodium salt ^‡H^
Chromium Note: soil determines food concentrations	Meats*^†‡ A^						Whole grains (possibly ^†‡^), barley, and oats	Grape juice and enriched drinks	
Copper	Organ meats*^†‡^	Beans, nuts^†^, seeds, and tofu	Shellfish^†^ and seafood^†^	Greek yogurt*^†‡^**	Avocado^‡^, figs, and prunes	Potatoes^‡^ and dark leafy greens^‡^	Wheat bran cereals (possibly ^†‡^) and whole grain products (possibly ^†‡^)		Dark chocolate^†‡^
Fluoride Note: the main source is the water supply * Amounts in food are minimal	Foods reconstituted with fluoridated water and fluoridated salt		Marine fish*^†‡^ and canned shellfish*^†‡^					Fluoridated water, water-based beverages, natural mineral waters > 1 mg/L labeled as “contains fluoride”, and tea	Toothpaste, dental products, mouth rinses
Iodine			Seafoods*^†‡^** and marine products *^†‡^	Cow’s milk and milk products*^†‡^** and egg yolk*^†^		Seaweed^H^	Products made with iodized salt		Iodized salt^H^; supplements
Manganese	Liver*^†‡ A^, and beef jerky*^†‡ A^	Nuts^†^ (e.g., hazelnuts, pecans, peanuts) Seeds (sesame) Soya beans, chickpeas, and lentils	Shellfish*^†‡^ (e.g., clams oysters, and mussels)		Fruit^‡^	Leafy vegetables^‡^ (spinach, kale)	Whole grains (possibly ^†‡^) and rice	Coffee^‡^ and tea	Chocolate^†‡^
Selenium Note: soil determines food concentrations	Meat*^†‡ A^	Brazil nuts^†^, sunflower seeds, lentils, and beans	Fish*^†‡^,	Milk*^†‡^**, cheese*^†‡^**, yogurt*^†‡^**, and eggs*^†^		Mushrooms	Cereals (possibly ^†‡^) (depending on soil content) Oatmeal, barley, and brown rice		Enriched foods
Zinc	Meat*^†‡ A^	Nuts^†^ (e.g. cashews, pecans, and peanuts); seeds (e.g., pumpkin^‡^, sunflower, and flaxseed), and legumes (e.g., lentils and beans)	Fish*^†‡^, oysters, crab, lobster, shrimp, and sardines (canned in oil, with bone)^†^	Eggs*^†^ and cheese^†^		Avocado^‡^	Grains and grain-based products (possibly ^†‡^) Fortified cereals (possibly ^†‡^) and oats	Oral rehydration solutions; vitamin/mineral drinks	Lozenges

*Restricted due to protein restriction; **restricted due to hyperlipidemia management

^†^Restricted due to phosphate restriction

^‡^Restricted due to potassium restriction

^A^Restricted due to hypervitaminosis A

^H^Restricted due to hypertension

References https://efsa.onlinelibrary.wiley.com/doi/epdf/10.2903/sp.efsa.2017.e15121; https://fdc.nal.usda.gov/fdc-app.html#/food-search?query=&type=Foundation

#### Evidence and rationale

When dietary modifications are needed in CKD, rich dietary sources of Mg and TEs may be reduced (Table 1). Food preparation may also alter Mg and TE contents (Table S4). Dietary intake of Mg and TEs has been reported to vary, ranging from above to below the requirements for healthy children [7–14] (Tables S5 to S22). Limited data suggests that children with advanced CKD (3b–5D) are at increased risk of malnutrition, which may be associated with low Mg and TE status. In diets with low animal-based protein, Zn intake may be reduced given that meats and seafood are major sources of Zn. According to a review paper,"I"intake may also be low due to CKD-required dietary restrictions of milk products, eggs, and iodized salt [15], which are the main sources.

In CKD, modifications to food preparation and cooking methods for the purpose of potassium or phosphate reduction may impact Mg and TE contents of foods. It is common practice to soak, boil, and drain green leafy vegetables, potatoes, root vegetables, and legumes to reduce potassium intake to manage hyperkalemia. This may also lower the food content of Cr, Cu, Mn, and Zn [16–24] (Table S4). Long periods of boiling and soaking in fluoridated water can increase Fl content [25]. Absorption and metabolism of Mg and TEs may also be altered by interactions with other nutrients (Table 2) [26–32].

4.0 What other factors influence magnesium and trace element status?

4.1 Consider level of kidney function, polyuria, dialysis modality, medical comorbidities, and medications when assessing and planning nutritional interventions specific to magnesium and trace element status.

4.1.1 Consider the risk of magnesium and trace element excess from exogenous sources.

**Table 2:** Magnesium and trace element interactions with medications and other nutrients

Nutrient	Interactions
Magnesium	*Major interactions*: Elvitegravir, baloxavir, bicetgravir, and eltrombopag — decreased exposure of drug *Moderate interactions*: Isradipine and nicardipine — hypotension Quniolones — decreased absorption *Mild interaction*: Bardoxolone — decrease in magnesium (intracellular shift) Citrate — heparin (nadroparin anticoagulation) in HD — increase in magnesium and calcium Proton pump inhibitors — decreased absorption Loop diuretics — increased excretion Tacrolimus — increased urinary losses
Chromium	*Moderate interaction*: Levothyroxine — decreased levothyroxine absorption Ascorbic acid and prostaglandin inhibitors (aspirin) — increased absorption Antacids agents and oxalate — decreased absorption Co-administration of Cr, Cu, Zn, and Fe — decreased intestinal uptake of other nutrients
Copper	Ascorbic acid — coadministration lowers copper absorption Penicillamine — copper (and iron) chelator, and increasing copper excretion Co-administration of Cr, Cu, Zn, and Fe — decreased intestinal uptake of other nutrients
Fluoride	No drug interactions High calcium increases fluoride absorption
Manganese	No known serious drug interactions May interact with quinolone or tetracycline antibiotics (low/serious risk) May interact with erythropoietin, ferrous sulfate, and calcium phosphate, which can decrease manganese absorption Low levels of manganese + iron/calcium — increased manganese absorption
Iodine	No known drug interactions Interaction with herbal supplements containing “bugleweed” may result in low iodine uptake
Selenium	Major interaction: Bictegravir and eltrombopag — decreased drug exposure/concentration Fiber — decreased Se in high-fiber diets
Zinc	*Major interaction*: Eltrombopag, elvitegravir, baloxavir, bictegravir — decreased drug exposure/concentration *Moderate interactions*: Iron — decreased gastrointestinal absorption of iron and/or zinc Quinolones — decreased quinolone effectiveness Penicillamine — decreased absorption/action of penicillamine Copper — decreased gastrointestinal absorption of copper and/or zinc Magnesium — decreased magnesium absorption with high supplementation doses Calcium — decreased net zinc absorption and balance with high dietary intake Thiazide diuretics — increased zinc excretion in the urine *Unknown level of interaction*: Deferiprone — decreased drug absorption with oral zinc Soy protein — increased bioavailability of zinc Fiber — decreased in Zn high-fiber diets Plant-based diet — decrease absorption Ascorbic acid supplementation — decreases bioavailability Enteral formula in food — decreases bioavailability Feed thickeners (guar gum) — reduced bioavailability Sodium polystyrene sulfonate — decreased zinc Co-administration/ingestion of Cr, Cu, Zn and Fe — decreased intestinal uptake of other nutrients Calcium fortification of milk — reduced bioavailability

#### Evidence and rationale

All children may develop Mg and TE deficiencies due to malabsorption or malnutrition, although evidence in children with CKD is limited [33, 34]. In CKD, the losses of protein-bound TEs (e.g., Cu, Se, and Zn) are affected by the degree of protein losses [35].

Absorption and metabolism may be altered by interactions with medications (Table 2). The most commonly prescribed medications for consideration are diuretics, phosphate binders, potassium binders, immunosuppressive agents, and formula thickeners. For example, the use of phosphate or potassium binders may decrease bioavailability and blood concentrations of Mg, Cu, Mn, and Zn in children [36–42] (Table S4). In infant formulas, the use of thickeners such as guar gum may decrease the bioavailability of Zn [43, 44]; the use of the phosphate binders sevelamer carbonate or sevelamer hydrochloride, or the potassium binders sodium polystyrene sulfonate or patiromer may lower the Mg, Cu, Mn, and Zn contents [37–42, 45]. However, in one study in adults on hemodialysis (HD), the choice of phosphate binders did not affect blood concentrations of Cu, Se, and Zn [46]. A meta-analysis in adults showed there is no evidence for a Mg lowering effect using the potassium binder sodium zirconium cyclosilicate [47].

#### Magnesium

Mg homeostasis is dependent upon adaptive changes to intestinal absorption and kidney excretion. According to classical studies in adults, urinary Mg excretion decreases as kidney function declines [48]. When the glomerular filtration rate (GFR) falls to < 30 ml/min/1.73 m^2^, homeostasis may no longer be maintained and hypermagnesemia is common, especially when GFR is < 10 ml/min/1.73 m^2^ [49]. A study in adults with CKD2–5 found that proteinuria may increase urinary Mg loss and increase the risk of Mg depletion [50]. Intestinal Mg absorption is primarily determined by dietary Mg intake [51].

Conversely, children on dialysis have been found to have high blood concentrations of Mg despite low dietary intakes of Mg or negative Mg balance [9, 14, 52, 53]. Children receiving peritoneal dialysis (PD) or with no urine output had higher blood concentrations than children on HD or producing urine [12, 14]. In PD, lower dialysis fluid Mg concentrations increase Mg removal and thus may lower blood Mg concentrations in children [52] and adults [54]. Mg is removed by HD in children with acute Mg toxicity [55]. The amount of removal is influenced by the dialysate Mg concentration and the blood concentrations of substances that bind Mg and thus decrease clearance (e.g., albumin and anions such as bicarbonate, citrate, and phosphate), as reported and reviewed in adults [56–60].

Medications may have a significant effect on Mg status (Table 2). Calcineurin inhibitors increase urinary Mg losses in children [9, 61] and adults [62] by inhibiting the Mg channel TRPM6 [9]. A similar mechanism explains the increased Mg losses with proton pump inhibitors [63]. The potassium-binding resin sodium polystyrene sulfonate also binds Mg, but the impact on serum Mg is less clear [37, 64]. The potassium binder patiromer also binds Mg and decreased serum Mg in adults receiving chronic HD [65]. There is conflicting evidence that increased dietary calcium (e.g., calcium supplements) decreases intestinal absorption of Mg, theoretically by competing with Mg for absorption [66]. Older studies in children and adults with CKD demonstrated increased absorption of Mg with calcitriol supplementation, although the clinical significance is uncertain [67].

The bioavailability of Mg-containing medication, such as phosphate binders (Mg hydroxide and Mg carbonate), is not known, but these may contribute to Mg intake in children with CKD [68].

#### Chromium

There is evidence of high blood Cr concentrations in children with pre-dialysis CKD [69] and in adults receiving PD and HD [70, 71]. Mass balance experiments in adult populations demonstrate a net gain of Cr during HD [71] and continuous ambulatory PD [72]. Elevated Cr levels may be associated with lactate-based (non-biocompatible) PD fluids, however, one single center study in pediatric patients suggests that this potential effect may be ameliorated through use of biocompatible fluids [69].

#### Copper

Low blood Cu may reflect decreased dietary intake, malabsorption, increased requirements, decreased plasma carrier proteins, or increased urinary losses, as reviewed in adults [73]. In one study in children with CKD2–5, blood Cu concentrations were normal in those receiving diet alone, oral nutritional supplements, or enteral tube feeds [74]. In another pediatric study, blood Cu concentrations were low in 17% of children on PD and HD despite dietary intake being above the reference range [14]. Whereas another study in children also demonstrated low blood Cu concentrations in PD and HD [75], a subsequent pediatric study found similar Cu concentrations in HD patients, pre-dialysis CKD patients, and controls [76].

#### Fluoride

There is a lack of data on Fl status in children in recent years. Fl deficiency is rare in CKD, although there is historical evidence to suggest FI excess in children with CKD [77, 78]. Urinary Fl excretion decreases as GFR declines [77, 79]; according to one study in infants and toddlers receiving PD, blood Fl can be elevated [78], though this is rarely evaluated in practice (expert opinion). There is minimal Fl removal with PD relative to urinary excretion by healthy kidneys [78]. Infants and toddlers receiving PD have higher blood Fl concentrations than healthy controls [78]. There is no data regarding Fl removal by HD. Clinicians should be aware of the Fl content of water (if available from water companies or health authorities) [80, 81], since polyuric infants and children drinking large amounts of fluoridated water (and having their formula reconstituted with fluoridated water) may be at risk of excess Fl intake and elevated blood Fl concentration, especially if they also have a decreased GFR. In all children, Fl excess may occur secondary to Fl supplements, excessive swallowing of Fl-containing toothpaste, and dental Fl treatments; in children with CKD with impaired clearance accumulation could be accelerated [77–79].

#### Iodine

According to historical and more recent studies in adults,"I"accumulates with decreasing GFR [82–84]. It has been shown that in acute “I” toxicity in adults;"I"is cleared during HD [85–87]. In the limited pediatric data, case studies have reported children being at risk of exogenous"I"excess from PD catheter cleaning solutions [88–91] and"I"injection when undergoing cystography [92]. In a study in"I"-deficient adults, goiter was more common in those with CKD than in healthy controls [93]**.** There are no studies reporting"I"biochemical status in children. Old studies in adults with CKD [94–96] and on HD [96, 97] have reported high blood concentrations, while newer studies in those receiving HD report blood concentrations within the normal range [98, 99].

#### Manganese

There is conflicting data on Mn concentrations in adults and children receiving dialysis. In adult meta-analysis [100] and studies in CKD2–5 and HD [101] and pre-dialysis patients [102], Mn levels may be low or elevated. In a cross-sectional study of children receiving PD and HD, Mn blood concentrations varied from normal (60%) to elevated (40%) [103]. In contrast, another pediatric study reported low concentrations in 14% of the children [104].

#### Selenium

There is limited literature regarding Se status in children with CKD, with studies demonstrating both lower blood concentrations in CKD2–5 [105] and HD and PD [105, 106], and similar blood concentrations in CKD2–5 [74] and in HD and PD [14, 103] compared to healthy controls. Since Se is protein-bound, losses may increase with increased urinary protein, as found in nephrotic syndrome, where blood Se concentrations were lower than in healthy controls in one single case–control study of 30 children [107]. Se losses with lower degrees of proteinuria have not been studied.

Low blood Se concentrations have been reported in children with CKD2–5 [74, 108] and HD and PD [108] and adults receiving HD [109, 110]. From the most recent available evidence in children with CKD, there appears to be no measurable loss of Se in the dialysis effluent during HD [111] or PD [112]. It is postulated that the low blood concentrations may reflect the redistribution of blood Se due to dialysis-induced inflammation rather than true removal of Se [113].

In one adult post-transplant study, the use of mycophenolate mofetil (MMF), a commonly used immunosuppressant, is associated with lower blood Se concentrations compared to non-MMF immunosuppressive regimens [114]. This may be related to increased gastrointestinal losses (expert opinion).

#### Zinc

GFR, urine output, dialysis modality, and medication interactions affect Zn status (Table 2). A 1986 study in children that included patients receiving dialysis, kidney transplant recipients, and healthy controls showed decreasing Zn excretion with decreased GFR [115].

There is conflicting information on the effect of dialysis on Zn status. Older studies from the 1980’s report Zn absorption in children during PD [13] and HD, likely due to water contamination [115–117] while there are three more recent reports of minimal losses in children on PD and continuous veno-venous HD [118] and more substantial losses of Zn during hemodiafiltrations in adults [111]. Of the three more recent studies in this area, there was conflicting evidence. One paper from 2009 [118] showed minimal Zn losses in children receiving PD and HD. In comparison, a 2022 study reported substantial losses of Zn during HD in adults [111]. In children receiving PD, Zn losses may be due to protein losses in the dialysate. The overall impact of dialysis on Zn status is complicated, with the possibility of losses due to diffusion, in addition to the losses of Zn bound to protein.

Low blood Zn concentrations have been observed with lower GFR. Rates of deficiency range from 30 to 60% in children on HD [52], PD and HD combined [103], PD [118], and with CKD [74, 119], although high concentrations have also been reported in children receiving PD or HD [14, 103]. Blood Zn concentrations have been lowest in children on dialysis, followed by CKD2–5 [120] when compared to healthy controls [76, 108, 121, 122]. In advanced CKD, the discrepancy between decreased urinary excretion with declining kidney function and more frequent Zn deficiency may be more reflective of lower dietary intakes with advancing CKD. Blood Zn concentrations may decrease due to urinary protein losses since Zn is bound to many proteins. One study in children with idiopathic nephrotic syndrome showed a positive correlation between urinary Zn and protein losses [35]. Whether this can be extrapolated to children with CKD is not known. Further, malnutrition is an important risk factor for Zn deficiency in children [34].

1.2 Environmental factors (e.g., soil and water mineral content) may impact the magnesium and trace element content of the diet.

#### Evidence and rationale

Intake of TEs is affected by water and soil contents. For example, location and soil composition of staple crops plays a role in population-level risk assessment of Cr, Se, and Zn status [123–125]. Water supplementation is typically the main source of dietary Fl; hence, as shown in a study of adolescents, the Fl concentration of local water largely determines Fl intake [79].

5.0 How should we approach clinical assessment and monitoring?

5.1 Biochemical assessment

5.1.1 We suggest biochemical assessment of magnesium status in those with hypokalemia, hypocalcemia, and those with clinical signs or symptoms of hypomagnesemia or hypermagnesemia.
5.1.2 Routine biochemical assessment of trace element status is not indicated.
5.1.3 Consider trace element biochemical assessment if there are *signs and symptoms* of deficiency or excess.
  In clinical suspicion of iodine deficiency or excess, serum inorganic iodide is the preferred test in children with reduced GFR. Interpretation requires additional testing and endocrinologic evaluation.
  Consider assessment of zinc status when there is suboptimal growth.
  Consider evaluation of zinc and copper as potential contributing factors in the setting of erythropoeitin stimulating agent (ESA)-resistant anemia.
5.1.4 Consider magnesium and trace element biochemical assessment if there are *risk factors* for deficiency or excess.
  Consider the role of medications that may impact magnesium and trace element excretion and absorption, specifically calcineurin inhibitors that lead to magnesium wasting.
  Consider the contribution and bioavailability of magnesium-containing medication.
  Consider the impact of multi-mineral interactions on magnesium and trace element status and evaluate with use of any combination of zinc, copper, or iron supplements.
  Consider the risk of nutrient excess when using adult formula or adult nutrient supplement.
  In children with polyuria and polydipsia, consider risk of fluoride excess with well-fluoridated water.
  In children with proteinuria, consider increased risk of deficiencies of zinc, copper, selenium, and magnesium.
  In children receiving HD, consider increased risk of deficiencies of selenium, zinc, and copper.
5.1.5 Biochemical assessment of magnesium and trace element status should ideally be undertaken when fasted, when not acutely ill, and prior to a HD treatment.
  Measure markers of inflammation (e.g. C-reactive protein) when assessing trace element status.

#### Evidence and rationale

Table 3 describes the common methods used for biochemical assessment. Regular biochemical assessment is often necessary for Mg given the risk of deficiency or excess in children with CKD. Routine biochemical assessment of TEs is not recommended, but selective evaluation is indicated if there are specific indications, as detailed below, for individual TEs. Children with proteinuria, and those using multi-mineral supplements or adult formulas, may need biochemical assessment (Table 2). Markers of inflammation (e.g., C-reactive protein, CRP) should be measured when assessing TE status as an indicator of inflammation, which may cause redistribution of TEs in the body, causing misleading biochemical results [113].

**Table 3:** Symptoms of deficiency, excess, non-dietary sources, biochemical assessment, and interactions of magnesium and trace elements

Nutrient	Role in body	Symptoms of deficiency	Symptoms of excess	Non-dietary sources	Biochemical assessment
Magnesium	Cofactor for many enzyme systems. Involved in aerobic and anaerobic energy generation and in glycolysis, either as an enzyme activator or part of the Mg-ATP complex. Required for oxidative phosphorylation. Plays a role in regulating potassium and metabolism of calcium	*See *Table 4 Early hypomagnesemia: nausea/vomiting anorexia, muscular weakness/tingling/spasms, numbness, cramps, and cardiac arrhythmias Severe deficiency: seizures, coma, and confusion Long-term deficiency: linked with osteoporosis, coronary heart disease and hypertension, insulin resistance, and impaired insulin excretion (limited evidence)	*See *Table 4 Diarrhea, absent or diminished deep tendon reflexes, cutaneous flushing, nausea/vomiting, headache, somnolence, arrhythmias, and urinary retention	Dialysate, depending on concentration Magnesium-containing medication, e.g., potassium binders magnesium hydroxide and magnesium carbonate	Blood* magnesium Urine (less commonly) Saliva (less commonly)
Chromium	Not fully determined Efficacy of insulin in regulating the metabolism of carbohydrates, lipids, and proteins	Glucose intolerance, weight loss, and a metabolic encephalopathy-like confusional state	Genotoxic and carcinogenic		Blood* chromium
Copper	Acts as a cofactor in several enzyme processes, with actions including: antioxidant defense, mitochondrial ATP generation, collagen synthesis, hormone activation, coagulation, modifying gene expression, hemoglobin synthesis, connective tissue metabolism, bone development, immune function, and defense against antioxidants It is also a known acute phase reactant It is essential for wound healing and immune function The collagen cross-linking copper proenzyme lysyl oxidase plays a major role in wound healing	Sideroblastic anemia, retarded growth, osteoporosis, neutropenia, decreased pigmentation, myeloneuropathy, leukopenia, neutropenia, bone demineralization, and compromised immune function	Few toxic effects; Wilson disease and liver dysfunction	Copper salt-containing topical creams for burn treatments; acidic foods cooked in uncoated copper cookware	Blood* copper
Fluoride	Development and strengthening of tooth enamel, prevention of dental caries, and reduces acid of bacteria on teeth; stimulates new bone formation	Dental caries Potential impact on bone formation (limited evidence)	Fluorosis with abnormal enamel deposition/dental pitting High dose excess: nausea/vomiting, abdominal pain, periostitis, and skeletal fluorosis	Dental products such as toothpaste and mouthwash/other dental products	Blood, saliva, and urine
Iodine	Component of the thyroid hormones thyroxine (T4) and triiodothyronine (T3) which are involved in metabolic regulation throughout life During the fetal stage, infancy, and childhood, these hormones are crucial for growth and neural and cognitive development, e.g., myelinization, neural migration and differentiation, and gene expression	Goiter (can become toxic and multinodular hyperthyroidism) Maternal deficiency: intellectual disability (cretinism) Hypothyroidism, intellectual and motor developmental impairment Thinning hair, dry skin, and bradycardia	Hyperthyroidism Mild symptoms consist of gastrointestinal upset, nausea, vomiting, and diarrhea, which may progress to delirium, stupor, and shock. It is rarely fatal Acute kidney injury in severe toxicity Hypothyroidism, intellectual, and motor developmental impairment Goiter Tachycardia and irregular pulse, tremor, and sweating	Contrast media used in imaging/ Iodine-containing antiseptics via burns injuries and catheters	In healthy children urinary excretion of iodine In CKD blood concentration/thyroid hormone
Manganese	Acts as a cofactor in several enzyme processes, with actions including: glycosaminoglycan synthesis (necessary for bone, cartilage and connective tissue synthesis); urea formation; amino acid, lipid and carbohydrate metabolism; and is a co-factor for SOD (superoxide dismutase dysfunction) and may contribute to excess oxidative stress in uremia	Fleeting dermatitis, miliaria crystallina, poor growth, bone formation, skeletal defects, abnormal glucose tolerance and altered lipid and carbohydrate metabolism in children	Permanent neurological disorder with tremors, facial spasms, and difficulty walking Central nervous system including tremors, muscle spasms, tinnitus, hearing loss, and unsteady on feet. Mania, insomnia, depression, delusions, anorexia, headaches, irritability, lower extremity weakness, changes in mood, altered reaction times, and reduced hand–eye coordination		Blood* manganese
Selenium	Acts as a cofactor in several enzyme processes, with actions including: thyroid hormone metabolism, antioxidant defense, and immunological function	“Keshan disease” — progressive cardiomyopathy, cardiovascular disease with thyroid disease, also with myositis, Kashin-Beck’s disease (arthropathy)	Irritation of mucous membranes, pallor irritability, and indigestion		Blood* selenium
Zinc	Acts as a cofactor in several enzyme processes, with actions including: bone homeostasis and growth; carbonic anhydrase needed for acid–base balance; matrix-metalloproteinases needed for wound healing; gene expression oxidative stress defense; and hemoglobin synthesis	Clinical phenotype of acrodermatitis enteropathica; anorexia, hypogeusia, growth retardation, diffuse skin lesions with impaired wound healing, alopecia, frequent infections, hypogonadism, diarrhea, beau lines, and angular cheilitis	Few toxic effects. High intakes cause: nausea, dizziness, headaches, gastric distress, gastric hemorrhage vomiting, loss of appetite, and anemia Occasional neurological symptoms: sensory ataxia and myelopathy Reduced immune function and decreased HDL levels	Possible contamination in medical fluids Denture adhesive cream Lozenges	Blood* zinc

*Refer to your local laboratory for preferential methods of assessment (whole blood/serum/plasma)

#### Magnesium

Routine assessment of blood Mg should be considered in CKD in the setting of risk factors for low and high concentrations. Detection of hypomagnesemia is important since it is associated with cardiovascular disease and increased mortality as reviewed in adults [126], both in the general population and in patients receiving HD [127]. In one study in children on chronic HD, lower serum Mg levels were associated with vascular calcification [127]. One study has reported a negative impact upon bone disease in children receiving PD [128]. Another study has shown a correlation between dental calculus and salivary Mg in children with CKD [129]. Mg should be measured in children with risk factors for unexplained hypocalcemia or refractory, unexplained hypokalemia. As explained in a review, hypomagnesemia impairs PTH secretion, which may cause hypocalcemia, and thus measuring Mg is appropriate in patients with unexplained hypocalcemia [130]. In addition, hypomagnesemia may increase urinary potassium losses by causing potassium secretion into the tubular lumen via ROMK channels in the distal nephron and thus Mg should be measured and hypomagnesemia corrected if there is hypokalemia that is refractory to supplementation [130]. Similarly, Mg should be measured when taking a medication such as tacrolimus that may increase urinary Mg losses [62] as suggested by one study in children [131] (Table 2), or Mg-containing phosphate binders, in children with known kidney wasting of Mg, or in those with risk factors for severe hypermagnesemia especially if symptoms are present.

#### Chromium

Despite evidence of elevated Cr blood levels in pediatric CKD and transplant [69] and adult dialysis patients [71, 72], adverse consequences have not been reported. Hence, routine biochemical assessment in the absence of clinical signs of excess (Table 3) is not indicated.

#### Copper

Low blood Cu concentrations appear to be uncommon in children with CKD [74, 121, 132] and in those on dialysis [14]. As suggested by one study in children on PD [7], routine monitoring is not indicated. Still, it may be considered in those with poor dietary intake, or when there are signs of Cu deficiency or excess. Cu is an acute phase reactant; CRP should be measured to determine if inflammation is present as there is a risk of falsely elevated Cu concentrations at times of inflammation. It has been reported that Cu deficiency may be a cause of anemia, as reviewed in children with CKD [133], and a cause of ESA-resistant anemia in adults on HD [134]. The mechanisms behind this have been better described in the general population [135, 136].

#### Fluoride

Blood Fl is rarely measured in CKD [78]. Measurement of blood Fl may be considered in a child with advanced CKD and a high intake of Fl due to polydipsia with a large intake of fluoridated water or fluoridated products.

#### Iodine

Measurement of blood"I"is indicated in a subset of children with biochemical evidence of hypothyroidism or hyperthyroidism and no alternative explanation, especially if there are risk factors for low or excess intake."I"deficiency can cause hypothyroidism with high thyroid-stimulating hormone (TSH) and low thyroxine (T4) concentrations;"I"excess can cause hypothyroidism or hyperthyroidism with normal TSH and low/normal triiodothyronine/T4 [83].

#### Manganese

There are no specific indications for measuring Mn concentrations in the general population or patients with CKD. If measured, whole blood is recommended as this matrix is not affected by the acute phase response.

#### Selenium

There are no specific indications for measuring Se concentrations in the general population or in patients with CKD. Se status may be assessed by measuring blood Se, although inflammation can lower concentrations [113, 137]. Hence, it is advisable to measure CRP to identify Se concentrations that may be falsely low in the setting of inflammation.

#### Zinc

Although assessment of blood Zn may not reflect whole-body stores, it is the only biochemical measurement available [138]. A 2020 review in non-CKD preschool children shows that blood Zn decreases with increasing inflammation [139]. This has also been observed in CKD and is thought to be due to tissue redistribution of Zn to the liver, as reviewed in adult HD patients [138] and reported in a study in adults with CKD and on dialysis [140].

Biochemical assessment of Zn should be considered in children with poor growth [141] or ESA-resistant anemia [138, 142–145]. The positive effect of Zn supplementation to correct Zn deficiency in improving growth has best been described in meta-analyses in the general pediatric population [146, 147]. Thus, in the setting of CKD and dialysis, it would be reasonable to assess Zn status where clinical assessment indicates a potential Zn deficiency is present, and growth stunting cannot be attributed to nutritional intake or disease-related effects.

Elevated Zn concentrations have been described in case reports of both children on PD [142] and adults on HD [144, 145] with ESA-resistant anemia, as defined by the inability to maintain desired hemoglobin concentrations despite high doses of ESA. In these case reports, Zn supplementation-induced Cu deficiency resulted in ESA-resistant anemia. Improved ESA-responsiveness was attributed to discontinuation of supplementation and initiation of Cu supplementation [142, 143, 145].

5.2 Physical assessment

5.2.1 We suggest individualized evaluation for physical signs and clinical symptoms of magnesium and trace element deficiency or excess.

- Consider magnesium, copper, iodine, manganese, and zinc intake and status when there is poor growth.
- Consider iodine deficiency and excess in goiter and hypothyroidism.

#### Evidence and rationale

Table 3 outlines the clinical manifestations, including physical findings of Mg and TE deficiency and excess. Screening for these findings is recommended as routine care for children with a variety of chronic conditions, including CKD [148]; the frequency is determined by clinical judgment and the outcome of relevant dietary and biochemical assessments. Signs and symptoms can overlap with clinical manifestations of CKD (e.g., impaired growth, poor appetite, vomiting, diarrhea, increased fractures, and neurological impairment), so deficiency or excess of TEs as a possible explanation for the clinical findings, when present, should be considered.

In the presence of poor growth, biochemical assessment and evaluation of dietary and supplemental intake should be considered for Mg, Cu,"I", Mn, and Zn since deficiency may have a negative impact on growth [12, 146, 149].

Clinical manifestations of hypomagnesemia and hypermagnesemia are described in Table 4 [58, 150–152]. The risk of hyperthyroidism or hypothyroidism, possibly caused by “I” excess or deficiency, is greater in CKD than in the general population as seen in adults [82, 83, 93, 97, 153–155] and demonstrated in case reports in infants with CKD [92] and in children on PD [89]. Hence, clinical assessment should include a physical examination to evaluate for goiter and screening for signs and symptoms for hypothyroidism or hyperthyroidism.

**Table 4:** Cut-off values and symptoms of hypo- and hypermagnesemia

Magnesium blood concentration	Symptoms
Normal: 0.7–1.05 mmol/L (1.7–2.3 mg/dL)*	Not applicable
Mild hypomagnesemia: 0.57–0.69 mmol/L (1.4–1.6 mg/dL)* [158]	Often asymptomatic
Moderate to severe hypomagnesemia: < 0.57 mmol/L (< 1.4 mg dL)*	Most symptoms are due to secondary hypocalcemia, which may cause tetany, laryngospasm, or seizures. Same symptoms may occur with isolated hypomagnesemia Symptoms most likely with severe hypomagnesemia (< 0.41 mmol/L [< 1 mg/dL]) Secondary hypokalemia
Mild hypermagnesemia: 1.05–2.2 mmol/L (2.55–5.35 mg/dL)* [159]	Well-tolerated; usually asymptomatic
Moderate hypermagnesemia: 2.2–3.5 mmol/L (5.35–8.5 mg/dL)* [59]	Non-specific symptoms such as nausea, dizziness, weakness, hypotonia, and confusion ECG changes: prolonged PR interval, QRS complex, and QT interval
Severe hypermagnesemia:* > 3.5 mmol/L (> 8.5 mg/dL) [160]	Paralysis, lethargy Hypotension, flushing, heart block, and cardiac arrest

*Normal levels and consequent definitions low or high levels vary between laboratories

5.3 Dietary assessment

5.3.1 We suggest a comprehensive dietary review should include magnesium and trace element intake, when databases allow.

- In particular, assessment of magnesium and trace element intake is warranted if there are clinical signs or symptoms and/or biochemical evidence of deficiency or excess.

5.3.2 We suggest that the intake of magnesium and trace elements in children with CKD2–5D/T be assessed by diet history/diet records, including food and drink, formulas and nutritional supplements, and a review of medications, when information is available.

- Consider non-prescribed products and exogenous sources as potential contributors of trace elements, even though quantification is not possible.

#### Evidence and rationale

In children with CKD, insufficient dietary intakes of Mg, Cu, Se, and Zn have been found [7, 9–11, 14]; however, sufficient intakes of Mg,"I", Se, and Zn have also been reported [8, 10]. One study in children on HD or PD has shown that the risk of insufficiency is higher in children who are not supplemented with formula/nutritional supplements [14].

Most nutrient analysis program databases are incomplete for TEs, making quantification of intake unreliable. The databases generally contain sufficient information for natural sources of Mg, Se, and Zn; however, food additives are often not included. Methods of dietary assessment were previously described in a PRNT publication [5]. Tables S5 to S22 show findings of studies that have reported dietary intakes of Mg and TEs in children with CKD2–5D/T [7–14]. A complete dietary assessment should include intake from food, drink (including water), formulas, nutritional supplements, and medications [156], with consideration of the effects of food preparation, dietary modification, nutrient interactions, and malabsorption. For specific TEs, a food frequency questionnaire designed to assess the intake of a particular nutrient may be more reliable than a food record. However, validated food frequency questionnaires are not widely available as they are food-culture-specific. It is not possible to assess the Fl content of food or drinks since it is largely determined by the unknown Fl content of the water used in their preparation. Local water supply, Fl supplements, and dental products are the main contributors to Fl intake. It has been estimated, that 75% of total Fl intake is from Fl-containing drinking water [25].

#### Magnesium

A study in infants and children on dialysis reported dietary intake of Mg correlates positively with nutritional outcomes such as growth, fat mass, and weight [12]. Children with CKD2–5 [10], HD, and PD [11] or HD/PD combined [12, 14], and transplant [9] have low dietary intakes compared with recommended intakes and healthy controls [9–12, 14]. A study reported that children on PD or HD eating food, rather than taking enteral formulas, were less likely to meet age-specific recommended dietary intakes [14]. Two studies report that older children and adolescents were less likely to meet recommended intakes of Mg than younger children on PD [11] and transplant [9].

6.0 When is dietary modification or supplementation of magnesium and trace elements indicated?

We suggest:

6.1 Optimizing overall nutritional intake before considering supplementation with magnesium and trace elements.
6.2 Using age-appropriate formulas and nutritional supplements to avoid the risk of nutrient excess.
6.3 Supplementing with zinc, selenium, and copper may be indicated when there is clinical and/or biochemical evidence of deficiency.
6.4 Avoiding co-administration of zinc, copper, and/or iron due to the risk of competitive inhibition of intestinal absorption.
6.5 Avoiding high regular intake of seaweed in CKD due to its high iodine content.
6.6 Restricting dietary and supplementary iodine in iodine excess-induced thyroid dysfunction.
6.7 Adjusting dietary intake and supplementation of magnesium to maintain blood magnesium within a safe range, which includes asymptomatic modest elevations.
  6.7.1 Only consider magnesium supplementation in proven biochemical deficiency and not based on dietary intake alone.
  6.7.2 Acute, severe hypomagnesemia and hypermagnesemia can be life-threatening and require rapid medical intervention.
  6.7.3 Mild asymptomatic hypermagnesemia does not need to be corrected.
  6.7.4 Moderate and severe hypomagnesemia should be managed by the administration of magnesium salts.
  6.7.5 To reduce the risk of diarrhea, it is suggested to distribute the total daily dose of magnesium taken as two to three doses spread across the day.
  6.7.6 Changes in magnesium management should be based on trends of serial results rather than a single result.

#### Evidence and rationale

Patients with CKD are at increased risk of high or low Mg and TE status. Low status may be addressed by dietary interventions and supplements. High status may be addressed by dietary interventions and decreasing or eliminating the use of supplements and non-dietary sources.

#### Magnesium

Blood concentrations of Mg do not always reflect dietary intake; levels may be high despite low dietary intake as reported in children on PD or HD [14] and transplant [9]. Therefore, supplementation should be based on blood concentrations. Several in vitro, animal, and adult human studies have linked Mg supplementation, higher blood concentrations, and dietary intake with decreased risk of vascular calcification [157], lower cardiovascular and all-cause mortality, improved glucose and lipid metabolism, and decreased inflammation reported in HD [158] and CKD [159] and reviewed in CKD [160]. Evidence in pediatric CKD patients is scarce [127], and a randomized-controlled trial in adult CKD patients did not demonstrate clinical benefit [161].

One study of 60 children with CKD found an improvement in GFR with Mg supplementation [68]. In adult HD patients, low Mg concentrations in blood have been associated with an increased risk of muscle cramping as reported in a study [162] and reviewed in 2020 [163].

Gastrointestinal side effects are common with oral Mg supplementation as demonstrated in studies in adults with pre-dialysis CKD [161, 164], adults on HD [165], and reviewed in 2020 [163]. In pediatric transplant recipients, supplementation of Mg using soy protein was more effective than Mg salts in increasing blood Mg concentrations, with reduced gastrointestinal side effects [166]. A study in adults on modulating dialysate Mg concentration suggests it is an effective strategy for increasing blood Mg concentration, causes no notable side effects, and has a low risk of symptomatic hypermagnesemia [167], although it is rarely used in practice.

A study in adult transplant patients found that nearly half of recipients were prescribed Mg supplementation for low blood concentrations [62]. A further study in adult transplant patients has shown an association between hypomagnesemia and disturbed glucose metabolism [168]. Hypomagnesemia post-transplantation may increase the risk for the development of new-onset diabetes after transplantation in adults [169] and children [170, 171], possibly related to the role of Mg in insulin resistance, although the association is not evident in all pediatric studies [172]. Hypomagnesemia has also been implicated in the development of hypertension in adult transplant patients [168]. In a single blinded crossover trial of this population group receiving cyclosporine, oral Mg supplementation increased weight and blood potassium but did not reduce blood pressure or lipid concentrations [173].

#### Copper

Zn and Cu share intestinal transporters. This may explain the decrease in the Cu/Zn ratio following Zn supplementation [174]. According to a study in children on dialysis, blood concentrations may be low despite dietary intake of Cu above recommended intakes, suggesting a Cu supplement may be required [14]. Another small study in children on PD reported no effect on blood Cu concentrations with Cu supplementation as part of a multivitamin-mineral supplement [7].

#### Fluoride

Fl supplementation is not recommended. One study reported children to have lower Fl excretion than adults and in children with kidney insufficiency, Fl reabsorption increased to around 80% with advancing CKD [77]. Another study in children with CKD showed an increased risk of excess intake due to decreased Fl excretion as GFR decreases [78]. Excessive swallowing of Fl-containing toothpaste and dental Fl treatments should generally be avoided. Excess intake of tea as an additional source of Fl should be considered.

If the Fl content of local water is known to be very high, it may be necessary to use other water sources, such as bottled water, especially in children with polyuria and secondary polydipsia [80]. In areas where Fl is not added to the water supply some supplementation, e.g., 25% of the usual treatment dose for children without CKD, may be appropriate.

#### Iodine

"I"excess may occur in CKD due to decreased urinary excretion. High blood concentrations may occur due to high regular intake of seaweed, which has a very high"I"content. Adults with CKD2–5 [84, 175], on PD [155] and HD [154], and high"I"intake have a favorable response to dietary"I"restriction, including normalized TSH and decreased blood"I"concentration and thyroid gland volume. In excess, exogenous sources need to be considered first and cautious handling of PD catheter “I” caps, for example, is warranted [89].

#### Selenium

There are no supplementation studies in children with CKD, but supplementation to meet the requirements for healthy children is suggested where there is deficiency.

#### Zinc

A study in children with CKD2–5D has shown that Zn supplementation increases blood Zn concentrations [176]. Zn supplements should not be given at the same time as iron, Cu, or calcium supplements given the risk of decreased Zn absorption due to competitive inhibition. In one study in children with CKD, HD and PD, Zn supplementation (15 or 30 mg per day) improved Zn status, but 40% remained deficient [120]. Zn supplementation has been associated with improved growth, body composition and blood albumin, and decreased CRP in children with CKD and on HD and PD [120, 177]. In addition, a decrease in elevated leptin concentrations, a contributing factor to CKD-related anorexia, has been noted with Zn supplementation in children with CKD and on HD [177]. Zn supplementation has been suggested to diminish the inflammatory response among adult HD patients and thus could be beneficial in patients with kidney failure [178]. Excessive Zn supplementation induced hypocupremia leading to progressive anemia in a case study of a child on PD [142] and in adults on HD [144, 145].

#### Manganese and chromium

Mn and Cr supplementation is not recommended; however, there is no evidence that multivitamin-mineral supplements containing Mn or Cr should be avoided.

## Results of Delphi survey

Twenty-seven responses were received via an electronic Delphi survey, comprising 10 dietitians, 16 pediatric nephrologists, and 1 dietetic physician across 18 countries. Delphi respondents are listed under Acknowledgements as “Participants in Delphi survey.”

Of the clinical practice recommendation statements, overall, an 89.1% consensus was achieved with a “strongly agree” or “agree” response, 5.8% had a “neutral” response, 1.2% “disagree,” and 3.2% “strongly disagree” response. All statements met the stipulated 70% or higher level of consensus. The taskforce team carefully reviewed all the statements in light of these responses; none required significant change.

## Research recommendations

There is a need for well-designed longitudinal observational studies of the dietary intake and status of Mg and TEs over time, accompanied by studies designed to determine requirements for children with CKD. The most pressing need is to characterize the Mg and TE status in parallel with changing kidney function and concomitant comorbidities such as proteinuria, anuria, and polyuria to ascertain the relationship between Mg and TE status on long-term outcomes such as growth and cardiovascular and bone health. Research recommendations are detailed in Table S23.

## Conclusions

Mg and TEs have essential roles in body processes. CKD can alter intake and biochemical status and thus requirements may differ in children with CKD compared with their healthy peers. We present CPPs addressing clinical issues pertaining to Mg and TE status in children with CKD (2–5D/T) as a key aspect of their nutritional management. Ensuring adequate Mg and TE intake and status is essential for preventing deficiency and excess, as well as for supporting development and long-term health. Promoting consumption of rich dietary sources of most TEs is advised. Dietary modifications necessary for raised urea, phosphate, and potassium concentrations may reduce TE intake but should not compromise TE status. Table 5 provides the summary of CPPs for Mg and TEs.

**Table 5:** Summary of recommendations

*Question*	Statements
*1.0 What are the magnesium and trace element requirements?*	1.1 The magnesium and trace element requirements for most children with CKD should approximate those of healthy children of the same chronological age 1.2 For some children with CKD, magnesium and trace element requirements may be less or greater than those for healthy children
*2.0 What are the main dietary sources of magnesium and trace elements?*	2.1 Children with CKD may obtain sufficient intake of magnesium and trace elements from a varied diet, breast milk, infant formula, or a nutritionally complete enteral formula
*3.0 How do dietary modifications affect intake?*	3.1 Dietary modifications and food preparation methods may impact magnesium and TE intake
*4.0 What other factors influence magnesium and trace element status?*	4.1 Consider level of kidney function, polyuria, dialysis modality, medical comorbidities, and medications when assessing and planning nutritional interventions specific to magnesium and trace element status 4.1.1 Consider the risk of magnesium and trace element excess from exogenous sources 4.2 Environmental factors (e.g., soil and water mineral content) may impact the magnesium and trace element content of the diet
*5.0 How should we approach clinical assessment and monitoring?*	5.1 Biochemical assessment 5.1.1 We suggest biochemical assessment of magnesium status in those with hypokalemia, hypocalcemia, and those with clinical signs or symptoms of hypomagnesemia or hypermagnesemia 5.1.2 Routine biochemical assessment of trace element status is not indicated 5.1.3 Consider trace element biochemical assessment if there are *signs and symptoms* of deficiency or excess • In clinical suspicion of iodine deficiency or excess, serum inorganic iodide is the preferred test in children with reduced GFR. Interpretation requires additional testing and endocrinologic evaluation • Consider assessment of zinc status when there is suboptimal growth • Consider evaluation of zinc and copper as potential contributing factors in the setting of erythropoeitin stimulating agent (ESA)-resistant anemia 5.1.4 Consider magnesium and trace element biochemical assessment if there are *risk factors* for deficiency or excess • Consider the role of medications that may impact magnesium and trace element excretion and absorption, specifically calcineurin inhibitors that lead to magnesium wasting • Consider the contribution and bioavailability of magnesium-containing medication • Consider the impact of multi-mineral interactions on magnesium and trace element status and evaluate with use of any combination of zinc, copper, or iron supplements • Consider the risk of nutrient excess when using adult formula or adult nutrient supplement • In children with polyuria and polydipsia, consider risk of fluoride excess with well-fluoridated water • In children with proteinuria, consider increased risk of deficiencies of zinc, copper, selenium, and magnesium • In children receiving HD, consider increased risk of deficiencies of selenium, zinc, and copper 5.1.5 Biochemical assessment of magnesium and trace element status should ideally be undertaken when fasted, when not acutely ill, and prior to a HD treatment • Measure markers of inflammation (e.g., C-reactive protein) when assessing trace element status
	5.2 Physical assessment 5.2.1 We suggest individualized evaluation for physical signs and clinical symptoms of trace element deficiency or excess • Consider magnesium, copper, iodine, manganese, and zinc intake and status when there is poor growth • Consider iodine deficiency in goiter and hypothyroidism
	5.3 Dietary assessment 5.3.1 We suggest a comprehensive dietary review should include magnesium and trace element intake, when databases allow • In particular, assessment of magnesium and trace element intake is warranted if there are clinical signs or symptoms and/or biochemical evidence of deficiency or excess 5.3.2 We suggest that the intake of magnesium and trace elements in children with CKD2–5D/T be assessed by diet history/diet records, including food and drink, formulas, and nutritional supplements, and a review of medications, when information is available • Consider non-prescribed products and exogenous sources as potential contributors of trace elements, even though quantification is not possible
*6.0 When is dietary modification or supplementation of magnesium and trace elements indicated?*	We suggest: 6.1 Optimizing overall nutritional intake before considering supplementation with magnesium and trace elements 6.2 Using age-appropriate formulas and nutritional supplements to avoid the risk of nutrient excess 6.3 Supplementing with zinc, selenium, and copper may be indicated when there is clinical and/or biochemical evidence of deficiency 6.4 Avoiding co-administration of zinc, copper, and/or iron due to the risk of competitive inhibition of intestinal absorption 6.5 Avoiding high regular intake of seaweed in CKD due to its high iodine content 6.6 Restricting dietary and supplementary iodine in iodine excess-induced thyroid dysfunction 6.7 Adjusting dietary intake and supplementation of magnesium to maintain blood magnesium within a safe range, which includes asymptomatic modest elevations 6.7.1 Only consider magnesium supplementation in proven biochemical deficiency and not based on dietary intake alone 6.7.2 Acute, severe hypomagnesemia, and hypermagnesemia can be life-threatening and require rapid medical intervention 6.7.3 Mild asymptomatic hypermagnesemia does not need to be corrected 6.7.4 Moderate and severe hypomagnesemia should be managed by the administration of magnesium salts 6.7.5 To reduce the risk of diarrhea, it is suggested to distribute the total daily dose of magnesium taken as two to three doses spread across the day 6.7.6 Changes in magnesium management should be based on trends of serial results rather than a single result

## Supplementary Information

Below is the link to the electronic supplementary material.

Supplementary file1 (DOCX 156 KB)

## Abbreviations

CKD: Chronic kidney disease
CKD2–5D/T: Chronic kidney disease stages 2–5, dialysis, and transplantation
CPPs: Clinical practice points
Cr: Chromium
Cu: Copper
Fl: Fluoride
HD: Hemodialysis
“I”: Iodine
Mg: Magnesium
MMF: Mycophenolate mofetil
Mn: Manganese
PD: Peritoneal dialysis
PICO: Patient, intervention, comparator, and outcomes
Se: Selenium
TE: Trace element
Zn: Zinc
