# Supplementary material for: Assessment and management of magnesium and trace element status in children with CKD stages 2–5, on dialysis and post-transplantation: Clinical practice points from the Pediatric Renal Nutrition Taskforce
Source: Pediatr Nephrol. 2025 May 17;40(10):3301–23. doi: 10.1007/s00467-025-06759-5 (PMC12402044; doi:10.1007/s00467-025-06759-5)
Supplement: Supplementary file 1 — Supplementary file1 (DOCX 156 KB) [file 467_2025_6759_MOESM1_ESM.docx]

**Table S1.** Search term strategy used in the literature review

| 1  Kidney Disease  Kidney Failure  Renal Failure  Renal Insufficiency  Kidney Injury  Kidney dysfunction  Chronic Kidney Disease (CKD)  Chronic Kidney Failure (CKF)  Chronic Renal Failure (CRF)  End Stage Renal Disease  End Stage Renal Failure  Renal Replacement Therapy  Dialysis  Pre Dialysis  Peritoneal Dialysis  Hemodialysis  Haemodialysis  CAPD continuous ambulatory peritoneal dialysis  APD automated peritoneal dialysis  Uraemia  Uremia  Transplant | 2  Diet  Dietary  Nutrition  Food  Feed  Intake  Requirements  Nutritional support  Dietary management  Dietary advice  Dietary restriction  Dietary modification  Supplementation  Dietitian  Dietician  Enteral nutrition  Oral intake  Diet recall  Mixed diet  Formula  Supplement(s)  Assessment  Human breast milk  Bioavailability  Nutrient interactions  Medication interactions  Assessment  Serum  Plasma  Red blood cell  Food record  Food frequency questionnaire (FFQ)  24-h recall (24 hour)  Diet history  Insufficiency  Insufficient  Subclinical  Deficiency  Toxicity  Excess  Adequacy  Erythrocyte  Urinary excretion  Urinary  Status  Nutriture  Depletion  Repletion  Liver (storage)  Tissue concentration  Prothrombin time | 3  Chromium, Cr  Copper, Cu  Fluoride, Fl  Iodine, I  Magnesium, Mg  Manganese, Mn  Selenium, Se  Zinc, Zn  Minerals  Trace elements  Micro minerals  Macro minerals |
| --- | --- | --- |

**Search methods**

1980 – May 2024, English language

Medline / PubMed, Embase, Cochrane library, Cinahl, manual searching

Given the paucity of studies in this field, all publications, including meta-analyses, randomized controlled trials, prospective studies, retrospective studies, case studies (irrespective of patient numbers) have been considered.

Adult studies were used when pediatric studies were lacking.

**Table S2.** A summary of internationally published recommended dietary intakes of magnesium and trace elements

| ***Nutrient*** | Magnesium | | | Fluoride | | Copper | | Iodine | | | Chromium | Manganese | Selenium | | | Zinc | | |
| --- | --- | --- | --- | --- | --- | --- | --- | --- | --- | --- | --- | --- | --- | --- | --- | --- | --- | --- |
| **Organisation/Body** | **LRNI** | **EAR/RNI/RI/PRI/DRI** | **UL** | **EAR/RNI/RI/PRI/DRI** | **UL** | **EAR/RNI/RI/PRI/DRI** | **UL** | **LRNI** | **EAR/RNI/RI/PRI/DRI** | **UL** | **EAR/RNI/RI/PRI/DRI** | **EAR/RNI/RI/PRI/DRI** | **LRNI** | **EAR/RNI/RI/PRI/DRI** | **UL** | **LRNI** | **EAR/RNI/RI/PRI/DRI** | **UL** |
| **Recommendations:** | mg/d(mmol) | | | mg/d | | mg (ug)/d | | ug/d | | | ug/d | mg/d | ug (umol)/d | | | mg (umol)/d | | |
| **Age (months):** |  |  |  |  |  |  |  |  |  |  |  |  |  |  |  |  |  |  |
| **0 thru 4 months** | 30 (1.2) | 26-60 (1.7-2.5) | NA | 0.01 | 0.7-1.2 | 0.2-0.3 (200) | NA | 40 | 50-110 | 1000 | 0.2 | 0.003-0.012 | 4.0-5.0 (0.1) | 6.0-15 (0.1-0.19) | 45 (0.57) | 2.6 (40) | 2.0-3.3 (50-60) | 4.0 |
|  | 26-60 (1.2-2.5) | | | 0.01-1.2 | | 0.2-0.3 (200) | | 40-1000 | | | 0.2 | 0.003-0.012 | 4.0-45 (0.1-0.57) | | | 2.0-4.0 (40-60) | | |
| **4 thru 6 months** | 40 (1.7) | 26-75 (2.1-2.5) | NA | 0.01 | 0.7-1.2 | 0.3 (200) | NA | 40 | 60-110 | 1000 | 0.2 | 0.003 | 5.0 (0.1) | 6.0-15 (0.2) | 45-55 (0.57) | 2.6 (40) | 2.0-3.3 (50-60) | 4.0 |
|  | 26-75 (1.7-2.5) | | | 0.01-1.2 | | 0.3 (200) | |  | 60-1000 | | 0.2 | 0.003 | 5.0-55 (0.2-0.57) | | | 2.0-4.0 (40-60) | | |
| **7 thru 11 months** | 45 (1.9) | 54-80 (2.5-3.3) | NA | 0.4-0.5 | 0.9-1.8 | 0.22-0.4 (220) | NA | 40 | 60-130 | 1000 | 5.5 | 0.02-0.6 | 5.0-6.0 (0.1) | 10-20 (0.1-0.25) | 60-70 (0.76) | 3.0 (45) | 2.5-3.8 (60-75 | 5.0 |
|  | 45-80 (1.9-3.3) | | | 0.4-1.8 | | 0.22-0.4 (220) | | 40-1000 | | | 5.5 | 0.02-0.6 | 5.0-70 (0.1-0.76) | | | 2.5-5.0 (45-75) | | |
| **1 year** | 45-50 (1.9-2.1) | 60-170 (2.5-3.5) | 65 | 0.6-0.7 | 1.3-2.4 | 0.34-0.7 (260-340) | 1.0 | 40 | 65-90 | 200-1000 | 11 | 0.5-2.0 | 6.7 (0.1) | 10-25 (0.1-0.25) | 70-90 (1.1) | 3.0 (45) | 2.5-4.5 (60-75) | 7.0 |
|  | 45-170 (1.6-3.5) | | | 0.6-2.4 | | 0.34-1.0 (260-340) | | 40-1000 | | | 11 | 0.5-2.0 | 6.7-90 (0.1-1.1) | | | 2.5-7.0 (45-75) | | |
| **2 years** | 50 (2.1) | 60-170 (2.7-3.5) | 65 | 0.6-0.7 | 1.3-2.4 | 0.34-0.7 (260-340) | 1.0 | 40 | 65-90 | 200-1000 | 11 | 0.5-2.0 | 7.0 (0.1) | 15-25 (0.2-0.25) | 70-91 (1.1) | 3.0 (45) | 2.5-4.5 (60-75) | 7.0 |
|  | 50-170 (2.1-3.5) | | | 0.6-2.4 | | 0.34-1.0 (260-340) | | 40-1000 | | | 11 | 0.5-2.0 | 7.0-91 (0.1-1.1) | | | 2.5-7.0 (45-75) | | |
| **3 years** | 50 (2.1) | 60-170 (2.7-3.5) | 65 | 0.6-0.7 | 1.3-2.4 | 0.34-1.0 (260-340) | 1.0 | 40 | 65-90 | 200-1000 | 15 | 0.5-2.0 | 7.0 (0.1) | 15-25 (0.2-0.25) | 90-95 (1.1) | 3.0 (45) | 2.5-4.3 (60-75) | 7.0 |
|  | 50-170 (2.1-3.5) | | | 0.6-2.4 | | 0.34-1.0 (260-340) | | 40-1000 | | | 15 | 0.5-2.0 | 7.0-95 (0.1-1.1) | | | 2.5-7.0 (45-75) | | |
| **4 years** | 70 (2.9) | 76-250 (3.7-4.8) | 110 | 1.0-1.5 | 2.2-4.4 | 0.4-1.0 (340-440) | 2.0-3.0 | 50 | 65-100 | 250-1000 | 15 | 1.0-2.5 | 10 (0.1) | 20-30 (0.3-0.38) | 95-150 (1.9) | 4.0 (60) | 3.0-5.8  (75-100) | 12 |
|  | 70-250 (2.9-4.8) | | | 1.0-4.4 | | 0.4-3.0 (340-440) | | 50-1000 | | | 15 | 1.0-2.5 | 10-150 (0.1-1.9) | | | 3.0-12 (60-100) | | |
| **5 years** | 70 (2.9) | 76-250 (3.7-4.8) | 110 | 1.0-1.5 | 2.2-4.4 | 0.4-1.0 (340-440) | 2.0-3.0 | 50 | 65-100 | 250-1000 | 15 | 1.0-2.5 | 10 (0.1) | 20-30 (0.3-0.38) | 95-150 (1.9) | 4.0 (60) | 3.0-5.8 (75-100) | 12 |
|  | 70-250 (2.9-4.8) | | | 1.0-4.4 | | 0.4-3.0 (340-440) | | 50-1000 | | | 15 | 1.0-2.5 | 10-150 (0.1-1.9) | | | 3.0-12 (60-100) | | |
| **6 years** | 70 (2.9) | 76-250 (3.7-4.8) | 110 | 1.0-1.5 | 2.2-4.4 | 0.5-1.0 (340-440) | 2.0-3.0 | 50 | 65-150 | 250-1000 | 15 | 1.0-2.5 | 10 (0.1) | 20-35 (0.3-0.38) | 130-150 (1.9) | 4.0 (60) | 3.0-7.7 (75-100) | 12 |
|  | 70-250 (2.9-4.8) | | | 1.0-4.4 | | 0.5-3.0 (340-440) | | 50-1000 | | | 15 | 1.0-2.5 | 10-150 (0.3-1.9) | | | 3.0-12 (60-100) | | |
| **7 years** | 115 (4.7) | 100-300 (6.7-8.0) | 110 | 1.0-1.5 | 2.2-4.4 | 0.5-1.0 (340-440) | 3.0 | 50 | 65-150 | 300-1000 | 15 | 1.5-2.5 | 16 (0.2) | 21-40 (0.38-0.4) | 130-150 (1.9) | 4.0 (60) | 3.0-7.7 (75-100) | 12 |
|  | 100-300 (4.7-8.0_ | | | 1.0-4.4 | | 0.5-3.0 (340-440) | | 50-1000 | | | 15 | 1.5-2.5 | 16-150 (0.2-1.9) | | | 3.0-12 (60-100) | | |
| **8 years** | 115 (4.7) | 100-300 (6.7-8.0) | 110 | 1.0-1.5 | 2.2-4.4 | 0.5-1.0 (340-440) | 3.0 | 50 | 65-150 | 300-1000 | 15 | 1.5-2.5 | 16 (0.2) | 21-40 (0.38-0.4) | 150 (1.9) | 4.0 (60) | 3.0-7.7 (80-110) | 12 |
|  | 100-300 (4.7-8.0) | | | 1.0-4.4 | | 0.5-3.0 (340-440) | | 50-1000 | | | 15 | 1.5-2.5 | 16-150 (0.2-1.9) | | | 3.0-12 (60-110) | | |
| **9 years** | 115 (4.7) | 100-300 (6.7-8.0) | 110 | 1.4-2.0 | 5.0-10 | 0.5-1.3 (540-700) | 3.0-5.0 | 55 | 75-150 | 300-1000 | 21-25 | 1.5-3.0 | 16 (0.2) | 21-50 (0.4-0.51) | 130-280 (3.6) | 4.0 (60) | 5.0-7.7 (80-110) | 25-34 |
|  | 100-300 (4.7-8.0) | | | 1.4-10 | | 0.5-5.0 (540-700) | | 55-1000 | | | 21-25 | 1.5-3.0 | 16-280 (0.2-3.6) | | | 4.0-34 (60-110) | | |
| **10 years** | 115 (4.7) | 150-300 (6.7-8.0) | 110 | 1.4-2.0 | 5.0-10 | 0.7-1.3 (540-700) | 3.0-5.0 | 55 | 75-120 | 300-1000 | 21-25 | 1.5-3.0 | 16 (0.2) | 26-55 (0.4-0.51) | 180-280 (3.6) | 4.0 (60) | 5.0-7.7 (80-110) | 25-34 |
|  | 110-300 (4.7-8.0) | | | 1.4-10 | | 0.7-5.0 (540-700) | | 55-1000 | | | 21-25 | 1.5-3.0 | 16-280 (0.2-3.6) | | | 4.0-34 (60-110) | | |
| **11 years** | 180 (7.4) | 200-300 (9.5-11.5) | 350 | 2.0-2.3 | 5.0-10 | 0.7-1.3 (540-700) | 3.0-5.0 | 65 | 75-130 | 450-1000 | 21-25 | 1.6-3.0 | 25 (0.3) | 26-65 (0.51-0.6) | 180-280 (3.6) | 5.3 (80) | 5.0-11.1 (110-140) | 25-34 |
|  | 180-350 (7.4-11.5) | | | 2.0-10 | | 0.7-5.0 (540-700) | | 65-1000 | | | 21-25 | 1.6-3.0 | 25-280 (0.3-3.6) | | | 5.0-34 (80-140) | | |
| **12 years** | 180 (7.4) | 200-300 (9.5-11.5) | 350 | 2.0-2.3 | 5.0-10 | 0.7-1.3 (540-700) | 4.0-5.0 | 65 | 75-130 | 450-1000 | 21-25 | 1.6-3.0 | 25 (0.3) | 26-65 (0.51-0.6) | 280 (3.6) | 5.3 (80) | 5.0-11.1 (110-140) | 24-34 |
|  | 180-300 (7.4-11.5) | | | 2.0-10 | | 0.7-5.0 (540-700) | | 65-1000 | | | 21-25 | 1.6-3.0 | 25-280 (0.3-3.6) | | | 5.0-34 (80-140) | | |
| **13 years** | 180 (7.4) | 200-300 (9.5-11.5) | 350 | 2.0-2.3 | 5.0-10 | 0.7-1.3 (540-700) | 4.0-5.0 | 65 | 75-150 | 450-1000 | 21-25 | 1.6-3.0 | 25 (0.3) | 26-65 (0.51-0.6) | 280 (3.6) | 5.3 (80) | 5.0-11.1 (110-140) | 25-34 |
|  | 180-350 (7.4-11.5) | | | 2.0-10 | | 0.7-5.0 (540-700) | | 65-1000 | | | 21-25 | 1.6-3.0 | 25-280 (0.3-3.6) | | | 5.0-34 (80-140) | | |
| **14 years** | 180 (7.4) | 220-410 (9.5-11.5) | 350 | 2.2-2.3 | 5.0-10 | 0.8-1.5 (685-890) | 4.0-8.0 | 65 | 95-150 | 450-1000 | 24-35 | 1.6-3.5 | 25 (0.3) | 26-70 (0.6-0.7) | 230-400 (5.1) | 4.0-5.3 (80) | 5.0-11.1 (110-140) | 34-35 |
|  | 180-410 (7.4-11.5) | | | 2.2-10 | | 0.8-8.0 (685-890) | | 65-1000 | | | 24-35 | 1.6-3.5 | 25-400 (0.3-5.1) | | | 4.0-35 (80-140) | | |
| **15 years** | 190 (7.8) | 220-410 (10.3-12.3) | 350 | 2.2-3.0 | 7-10 | 1.0-1.5 (685-890) | 4.0-8.0 | 70 | 95-150 | 500-1000 | 24-35 | 1.6-3.5 | 40 (0.5) | 26-85 (0.7-0.9) | 230-400 (5.1) | 4.0-5.5 (60-85) | 5.0-14.2 (110-145) | 34-35 |
|  | 190-410 (7.8-12.3) | | | 2.2-10 | | 1.0-8.0 (685-890) | | 70-1000 | | | 24-35 | 1.6-3.5 | 40-400 (0.5-5.1) | | | 4.0-35 (60-145) | | |
| **16 years** | 190 (7.8) | 220-410 (10.3-12.3) | 350 | 2.2-3.0 | 7-10 | 1.0-1.2 (685-890) | 4.0-8.0 | 70 | 95-150 | 500-1000 | 24-35 | 1.6-3.5 | 40 (0.5) | 26-85 (0.7-0.9) | 230-400 (5.1) | 4.0-5.5 (60-85) | 5.0-14.2 (85-145) | 34-35 |
|  | 190-410 (7.8-12.3) | | | 2.2-10 | | 1.0-8.0 (685-890) | | 70-1000 | | | 24-35 | 1.6-3.5 | 40-400 (0.5-5.1) | | | 4.0-35 (60-145) | | |
| **17 years** | 190 (7.8) | 220-410 (10.3-12.3) | 350 | 2.3-3.0 | 7-10 | 1.1-1.5 (685-890) | 4.0-8.0 | 70 | 95-150 | 500-1000 | 24-35 | 1.6-3.5 | 40 (0.5) | 26-85 (0.7-0.9) | 255-400 (5.1) | 4.0-5.5 (60-85) | 5.0-14.2 (85-145) | 34-35 |
|  | 190-410 (7.8-12.3) | | | 2.3-10 | | 1.1-8.0 (685-890) | | 70-1000 | | | 24-35 | 1.6-3.5 | 40-400 (0.5-5.1) | | | 4.0-35 (60-145) | | |
| **18 years** | 190 (7.8) | 220-410 (10.3-12.3) | 350 | 2.9-3.4 | 7 | 1.1-1.8 (685-890) | 5.0-8.0) | 70 | 95-150 | 600-1000 | 24-35 | 1.6-3.5 | 40 (0.5) | 26-90 (0.7-0.9) | 400 (5.1) | 4.0-5.5 (60-85) | 5.0-13 (85-145) | 34-35 |
|  | 190-410 (7.8-12.3) | | | 2.9-7.0 | | 1.1-8.0 (685-890) | | 70-1000 | | | 24-35 | 1.6-3.5 | 40-400 (0.5-5.1) | | | 4.0-35 (60-145) | | |

Sources: DoH, ‘Dietary Reference Values for Food Energy and Nutrients for the United Kingdom’; IoM, ‘Dietary Reference Intakes for Vitamin A, Vitamin K, Arsenic, Boron, Chromium, Copper, Iodine, Iron, Manganese, Molybdenum, Nickel, Silicon, Vanadium, and Zinc.’; EFSA, ‘EFSA NDA Panel (EFSA Panel on Dietetic Products, Nutrition and Allergies), 2013. Scientific Opinion on Nutrient Requirements and Dietary Intakes of Infants and Young Children in the European Union.’; NNR, ‘Nordic Nutritional Recommendations 2023’; NHMRC, ‘Nutrient Reference Values for Australia and New Zeland. Including Recommended Dietary Intakes’.

**Table S3. Definitions of requirements.**

| **EAR** | **Estimated Average requirement** | **Average daily intake level to meet 50% of needs** |
| --- | --- | --- |
| **RNI** | **Recommended nutrient intake** | **EAR + 2 standard deviations (SD) to meet the need of most healthy individuals in age and sex-specific populations (97.5%)** |
| **AH** | **Apparently healthy** | **Absence of disease based on clinical signs and symptoms of micronutrient deficiency or excess and normal function on laboratory values and physical examination** |
| **PNI** | **Protective nutrient intake** | **Amount greater than RNI which may be protective against a specific health or nutrition risk of public health relevance** |
| **UL** | **Upper tolerable nutrient intake level** | **Maximum intake from food, water and supplements that is unlikely to pose a risk of adverse health effects from excess in most individuals.** |
| **NE** | **Nutrient excess** | **Above the highest level at which no observed adverse effects of biological significance are found** |
| **NOAEL** | **No observed adverse effect level** |  |
| **LOAEL** | **Lowest observed adverse effect level** |  |
| **OI** | **Optimal intake** | **Establish the function of interest and the desired level of function.** |

**Sources of terminology:**

**IoM, ‘Dietary Reference Intakes for Vitamin A, Vitamin K, Arsenic, Boron, Chromium, Copper, Iodine, Iron, Manganese, Molybdenum, Nickel, Silicon, Vanadium, and Zinc.’; DoH, ‘Dietary Reference Values for Food Energy and Nutrients for the United Kingdom’; EFSA, ‘EFSA NDA Panel (EFSA Panel on Dietetic Products, Nutrition and Allergies), 2013. Scientific Opinion on Nutrient Requirements and Dietary Intakes of Infants and Young Children in the European Union.’; NNR, ‘Nordic Nutritional Recommendations 2012’; NHMRC, ‘Nutrient Reference Values for Australia and New Zeland. Including Recommended Dietary Intakes’.**

**Table S4 Summary of cooking effects on magnesium and trace elements**

| **Mineral / Trace element** | **Cook and process** | | | | **Pretreated formula** | **Competitive Interactions** |
| --- | --- | --- | --- | --- | --- | --- |
|  | **Defrost / Process** | **Boil & Drain** | **Bake** | **Microwave** | **Pretreated formula** | **Bioavailability impacted** |
| **Chromium**  **(impacted by heat)** |  |  |  | **Broadbean seeds microwaved**  ***↓* 13-14% (Lisiewska 2008)** |  |  |
| **Copper**  **(soluble in water,**  **impacted by heat)** | **Lentils dehulled**  ***↓*16% (Wang 2009)** | **Legumes blanched**  ***↓* (Lisiewska 2008))**  **Lentils boiled**  **↑ 12%(Wang 2009)**  **Chickpeas boiled**  ***↓* 33.6% (Alajaji and El-Adawy, 2006)**  **Broadbean seeds blanched**  ***↓* 10%(Lisiewska 2008)**  **French bean  blanched**  ***↓* (Lisiewska 2008)**  **Rice boiled *↓*(Kimura 1990)** | **Chickpeas autoclaved**  ***↓* 26.4% (Alajaji and El-Adawy, 2006)** | **Chickpeas microwaved**  ***↓*   14.5% (Alajaji and El-Adawy, 2006)** | **Paediatric renal feed (Suplena) + SPS (Sodium Polystyrene Sulfonate) *↓*3-11% (Taylor 2015)**  **Paediatric renal feed + Sevelamer carbonate *↓* 19% (Taylor 2015)**  **Standard infant formula + SPS *↓* 5-19% (Taylor 2015)** | **Iron**  **(negatively) Perez-Llamas 2001**  **Calcium (negatively) Perez-Llamas 2001**  **Sevelamer Carbonate (negatively) Taylor 2015**  **SPS (negatively) Taylor 2015** |
| **Fluoride**  **(soluble in water, increased uptake with heat)** |  | **Increased in boiling and soaking ref Sawangjang** |  |  |  |  |
| **Iodine** |  |  |  |  |  |  |
| **Magnesium**  **(soluble in water)** | **Lentils dehulled**  ***↓*20% (Wang 2009)** | **Potatoes cubed & boiled**  ***↓* 65% (Bethke 2008)**  **Potatoes shred & boiled**  ***↓* 70% (Bethke 2008)**  **Lentils boiled**  ***↓* 10% (Wang 2009)**  **Chickpeas boiled**  ***↓* 6.3%**  **(Alajaji and El-Adawy, 2006)**  **Breadfruit boiled**  ***↓*  35.7%**  **(de Sousa 2016)**  **Mustard leaves boiled**  ***↓* 36%(Lima 2019)**  **French peas blanched – frozen – boiled**  ***↓*  17-31%**  **Compared with microwaving**  **(Lisiewska 2008)**  **Rice boiled *↓* (Kimura 1990)**  **Spinach boiled**  ***↓*  87% (Kimura 1990)** | **Chickpeas autoclaved**  ***↓*  2.8%**  **(Alajaji and El-Adawy, 2006)**  **Mustard leaves**  **baked**  ***↓* 60.6% (Lima 2019)**  **Spinach baked**  ***↓*51% (Kimura 1990)** | **Chickpeas microwaved**  ***↓* 1.7%**  **(Alajaji and El-Adawy, 2006)**  **Breadfruit**  **microwaved**  ***↓* 18.9%**  **(de Sousa 2016)**  **Mustard leaves**  **microwaved**  ***↓* 69.7% (Lima 2019)** | **Standard infant formula + SPS**  ***↓* 3-26% (Taylor 2015)**  **Standard infant formula + SPS**  **↔(Thompson 2013)**  **EBM + SPS**  **↔(Thompson 2013)**  **Renal complete feed (Suplena) +SPS**  **↔(Taylor 2015)**  **Paediatric renal feed + Sevelamer carbonate *↓* 19% (Taylor 2015)**  **Semi skimmed cows milk, breast milk, baby formula and tube feeding formula + sevelamer hydrochloride and sevelamer carbonate  *↓* 5-25% (Raaijmakers 2013)**  **Paediatric renal feed + SPS + Sevelamer**  **↑  (Taylor 2015)**  **Enteral high protein formula + Kayexalate**  **↔ (Rivard 2004)**  **Infant formula + patiromer *↓* 63-71% (Paloian 2019)**  **Enteral feeds + SPS**  **↔  (Le Palma 2018)** | **Fibre (negatively) Perez-Llamas 2001)**  **Patiromer (negatively) Paloian 2019**  **SPS (positively) Raaijmakers 2013**  **Sevelamer Carbonate (positively) Raaijmakers 2013**  **(negatively Taylor 2015)** |
| **Manganese**  **(soluble in water)** | **Lentils dehulled**  ***↓* 11.5% (Wang 2009)** | **Potatoes cubed & boiled *↓* 25-30% (Bethke 2008)**  **Potatoes shredded & boiled**  ***↓* 50% (Bethke 2008)**  **Legumes blanched**  ***↓* (Lisiewska 2008)**  **Lentils boiled**  ***↓* 6% (Wang 2009)**  **Chickpeas boiled**  ***↓* 14.7% (Alajaji and El-Adawy 2006)**  **Breadfruit boiled**  ***↓* 65.6% (de Sousa 2016)**  **Broadbean seeds blanched**  ***↓* 10% (Lisiewska 2008)**  **French bean  blanched *↓* (Lisiewska 2008)**  **Rice boiled**  ***↓*(Kimura 1990)** | **Chickpeas autoclaved *↓*10% (Alajaji and El-Adawy, 2006)** | **Chickpeas microwaved *↓* 3.8%  (Alajaji and El-Adawy, 2006)**  **Breadfruit microwaved**  ***↓* 31.2% (de Sousa 2016)** | **Paediatric Renal feed (Suplena) + SPS (Sodium Polystyrene Sulfonate)**  ***↓* 3-16% (Taylor 2015)**  **Paediatric renal feed + Sevelamer carbonate**  ***↓* 16% (Taylor 2015)**  **Paediatric renal feed + SPS + Sevelamer**  ***↓* (Taylor 2015)** | **SPS (negatively) Raaijmakers 2013, Taylor 2015**  **Sevelamer Carbonate (negatively) Raaijmakers 2013,**  **Taylor 2015** |
| **Selenium** |  |  |  |  |  | **Fibre**  **(Negatively) Perez-Llamas 2001)** |
| **Zinc**  **(soluble in water, impacted by heat)** | **Broadbeans frozen and stored 6 mths**  ***↓*(Kmiecik 2000)**  **Broadbeans frozen and cooked**  ***↓* 27% (Kmiecik 2000)**  **Broadbeans canned.**  ***↓*27% (Kmiecik 2000)** | **Potatoes cubed & boiled *↓* 25-30% (Bethke 2008)**  **Potatoes shredded & boiled**  ***↓* 50%  (Bethke 2008)**  **Legumes blanched**  ***↓* (Lisiewska 2008)**  **Lentils boiled**  ***↓* 8%(Wang 2009)**  **Chickpeas boiled**  ***↓* 20.8%**  **(Alajaji and El-Adawy, 2006)**  **Mustard leaves boiled**  ***↓* 46%  (Lima 2019)**  **Broadbean seeds blanched**  ***↓* 10% (Lisiewska 2008)**  ***↓* 19% (Kmiecik 2000)**  **French bean  blanched *↓*(Lisiewska 2008)**  **Spinach boiled.**  ***↓*78% (Kimura 1990)** | **Chickpeas autoclaved *↓*10%**  **(Alajaji and El-Adawy, 2006)**  **Mustard leaves baked  *↓*61.5% (Lima 2019)**  **Spinach baked**  ***↓*34% (Kimura 1990)** | **Chickpeas microwaved *↓*8.6%**  **(Alajaji and El-Adawy, 2006)**  **Mustard leaves microwaved *↓*61.5% (Lima 2019)** | **Paediatric renal feed (Suplena)  + SPS (Sodium Polystyrene Sulfonate)**  ***↓* 5-20% (Taylor 2015)**  **Paediatric renal feed + Sevelamer carbonate *↓* 20% (Taylor 2015**  **Standard infant formula + SPS had no affect↔ (Taylor 2015)**  **(Fassinger 1998)**  **Paediatric renal feed + SPS + Sevelamer**  ***↓* (Taylor 2015)**  **Enteral high protein formula + Kayexalate**  **↑  33% (Rivard 2004)** | **Iron (negatively) Hemalatha 2009**  **Calcium (negatively) Hemalatha 2009, Perales 2006**  **Guar gum**  **(Negatively) Bosscher 2001, 2003**  **Soy protein**  **(Positively) Hemalatha 2009**  **Fibre (negatively) (Van Dyck 96, Perez-Llamas 2001)**  **Ascorbic Acid (negatively) (Van Dyck 96)**  **NaCl (positively) Hemalatha 2009**  **Kayexalate (positively) Rivard 2004**  **SPS (negatively) Raaijmakers 2013, Taylor 2015**  **Sevelamer Carbonate (negatively) Raaijmakers 2013**  **Taylor 2015** |

**↔ no change, ↑ increase, *↓ decrease***

**Abbreviations used in tables S5-S22:**

CKD, chronic kidney disease

CNI, Calcineurin inhibitors

Cr, Chromium

Cu, Copper

DRI Dietary Reference Intake

EER Estimated Energy Requirement

EGF excretion fraction

ESKD end stage kidney disease

Fl, Fluoride

I, Iodine

IQR Inter Quartile Range

HD Hemodialysis

Mn, Manganese

Mg, magnesium

Mo, Months

PD Peritoneal dialysis

PTH Parathyroid Hormone

P-Pi Plasma phosphate

Tx Kidney Transplant

RDI Recommended Dietary Intake

Se, Selenium

U urea

Yrs, Years

Zn, zinc

**Table S5. Mg: dietary Intake in pediatric studies**

| **Author, year, & location** | **Type of study** | **Patients** | **Age** | **N** | **Intervention** | **Outcome measures** | **Findings** | **Potential bias / limitations** |
| --- | --- | --- | --- | --- | --- | --- | --- | --- |
| Ledeganck 2018, Belgium | Longitudinal, observational | Nephrotic syndrome and post kidneyTx treated with CNI and non-CNI treated children | 2.3- 20.3 yrs | 23 post Tx + 24 CKD | Comparison of Mg intake, serum levels and excretion levels between children with nephrotic syndrome and children post Tx treated either with CNI (calcineurin inhibitor) and non-CNI treated. | Mg intake from Food Frequency Questionnaire; serum Mg, urinary Mg, creatinine | Median intake Mg: post Tx with CNI =89% RDI (Range 37-684) with 27.3% > RDI.  CKD without CNI= 86% RDI (range 63-436) with 30.4%> RDI  Mg intake correlated with age: % >RDI for <8y:63.2%; 8-12 yr: 17.9%; 12-16 yr: 33.3%; >16yr: 21.1%.  Use of Mg supplements was negatively correlated with Mg intake.  No correlation of Mg intake with BMI z , sex, serum Mg levels, kidney function, serum or urinary EGF concentration or the presence of hypomagnesemia. | A limitation was that CNI treated patients were included when they were already on treatment with CNI.  Study main comparison was Mg intake/ serum and excretion with or without CNI treatment. Difficult to compare CKD stages eg. CKD / kidney Tx |
| Lindeback 2023, Australia | Cross-sectional | CKD | 2-18 yrs | 36 | Nutritional intake and diet quality of children with CKD compared with controls. | Intake of macronutrients, micronutrients and diet quality, food frequency,  Weight, Height, Body Mass Index | Children with CKD were significantly less likely to meet requirements for Mg intake than healthy controls.  Mean Mg intake was 379 mg/day (+-131) (180% RDI) for children with CKD and 395 mg/day ( +-140) (217% RDI) for controls.  69.4% of children with CKD met RDI for Mg (25/36).  92.7% of healthy controls met RDI for Mg (76/82). | Small sample size.  Sample did not include children on dialysis.  Sample included children with CKD pre Tx and post Tx.  Children with <80% EER from oral diet excluded. |

| My Thuc 2019, Vietnam | cross-sectional | PD | 2-15 yrs | 31 | Dietary recall | Dietary intake via a 24 hour recall. Nutrition analysis. | Mg intake ranged from 34-120% of DRI.  Daily intake of Mg for children under the age of 10 met over 92% RDI.  Daily intake of Mg of over 10 yr old children was less than 67% of RDI’s. | Small sample. Population had high incidence of malnutrition (35.5%).  Serum Mg was not measured.  Intake from dialysate not measured.  Compared with usual recommendations for dietary reference intakes for the Vietnamese general population. |
| --- | --- | --- | --- | --- | --- | --- | --- | --- |
| Pontón-Vázquez 2017, Mexico | cross-sectional | ESKD | 13.6 yrs +- 2 yrs | 55 total  22 PD,  33 HD | Comparison of dietary intake, anthropometry and body composition in PD and HD.  . | 2 x 24-hour dietary recall.  Anthropometric, biochemical and body composition indicators- Mid Upper Arm Circumference, EUC, serum glucose, lipids, intakes of Vit C and Na. | Children on PD whose Mg intake <120% adequacy were significantly more likely to have fat mass <80% of the age and gender related median.  For children on PD, adequacy of Mg intake was significantly associated with Mid Upper Arm Circumference z score and Arm Muscle Area. |  |
| Tuokkola 2021, Finland  . | Retrospective cross-sectional | Children on PD / HD | 0.8 - 8.3 yrs (median = 1.16) | 3 | PD/HD vs recommended intakes / normal reference ranges | Dietary intake (solids + enteral) and blood concentrations. | Median Mg intake was 87.8mg (IQR 78.0 - 102.9) mg/day = 98% Recommended. intake (IQR 74-110%)  . | No control groups. |

**Table S6. Mg: biochemical status in pediatric studies**

| **Author, year, & location** | **Type of study** | **Patients** | **Age** | **N** | **Intervention** | **Outcome measures** | **Findings.** ± | **Potential bias / limitations** |
| --- | --- | --- | --- | --- | --- | --- | --- | --- |
| Aksoy 2020, Turkey | Retrospective | Kidney Tx without diabetes mellitus | 21-259 Mo  (mean 157±55 Mo) | 188 | Retrospective evaluation of serum Mg and glucose levels from medical records and new onset diabetes mellitus | Serum Mg and glucose levels 1 Mo post Tx  Development of new onset diabetes mellitus | New onset diabetes mellitus diagnosed in 7 patients (3.7%). Hypomagnesemia was shown in 50% of patients (64/120).  Mean glucose levels higher in those with hypomagnesemia (119.2mg/dL) than in patients without hypomagnesemia (91.56mg/dL), P= .01.  A significant negative correlation was observed between serum Mg and glucose levels (r = -0.53, P<.05).  Mean serum Mg level in patients with new onset diabetes mellitus was lower than in patients without diabetes (1.56 ± 0.21 vs 1.75 ± 0.26mg/dL; P = .052) | Mg supplementation was not documented. |
| El Tayeb 2009, Egypt | Cross-sectional | Children on HD | 9-17 yr | 24 |  | Weight, Height, Body Mass Index, blood pressure, Serum Mg, Zn, PTH, Ca, PO, Alkaline phosphatase, Urea, Creatinine | Mean serum Mg in children with CKD on Dialysis 0.88 (mmol/L) ± 0.27. Was significantly higher than Mean serum Mg in healthy control children 0.65 mmol/L ± 0.19 (P <0.01).  Insignificant negative correlation between PTH and serum Mg in CRF pts. Lower serum Mg levels were insignificantly correlated with higher PTH., (r = -0.3047, p = 0.1478) |  |
| Freundlich 1985, USA | Case study | PD | 1 week - 16 yrs (mean 7 mo) | 7 | Peritoneal fluxes of minerals and bone modulating hormones and their impact on corresponding serum levels and bone mineralization were studied | Serum Mg, Ca, PO, Protein  Bone mineral content (BMC) | Initial Hypermagnesemia (3.6 ±0.6mg/dL) declined in all patients (3.3 ± 0.9mg/dL) but normalized in only 1 patient.  Average daily removal of 18mg Mg.  Initial bone mineral content in 5 patients revealed significant undermineralization, which stabilized / remineralised in 2 patients on follow up.  Peritoneal losses of Mg although substantial, were not sufficient to normalize hypermagnesemia in most patients. | Small study with multiple measures per patient on multiple dialysis cycles. |
| Gökceoğlu 2014, Turkey | Retrospective | Tx | Mean age 13.6 yrs ± 3.7 | 91 | Retrospective chart review of serum Mg and urinary Mg. | Serum Mg, eGFR | Patients with higher tacrolimus trough blood levels lower GFR and early post Tx period had risk of hypomagnesaemia.  Mean serum Mg levels 1.73 ± 0.22 mg/dL.  Mean serum Mg of patients with and without oral Mg replacement were 1.68±0.20mg/dL and 1.76±0.22 mg/dL (p=0.236). Mean Fraction Excretion Mg levels of patients with and without oral Mg replacement were 5.94 ± 3.65% and 5.91 ± 3.72% (p=0.869).  38 patients (41%) had hypomagnesemia.  Negative correlation between serum Mg levels and estimated GFR and blood tacrolimus trough levels (r = -0.215, p = 0.040 and r = -0.409, p = 0.000, respectively).  Positive correlation between serum Mg levels and Tx duration (r=0.249, p=0.017). Mean fractional Mg excretion was 5.9.± 3.7% and 59 patients (65%) had high Mg excretion.  Significant negative correlation between fractional Mg excretion and estimated GFR (r=0.432, p=0.001).  Significant positive correlation between fractional Mg excretion and blood creatinine (r=0.379, p=0.003). |  |
| Hayes 2017, UK | Retrospective | Tx | 7 rs (range 1.3-7.5) yrs | 173 | Retrospective analysis of risk factors for new onset diabetes after Tx | Serum Mg | 5 (3%) had sustained hypomagnesemia.  Hypomagnesemia is a significant independent risk factor for the development of new onset diabetes after Tx in pediatric kidney Tx patients. | Further studies needed to clarity the etiology of this association and examine effect of Mg supplementation on new onset diabetes after Tx. |
| Ledeganck  2018, Belgium | Longitudinal, observational | Nephrotic syndrome and Tx | 2.3 - 20 y | 23 post kidney Tx + 24 CKD | Comparison of Mg intake , blood Mg, excretion level treated with CNI (calcineurin inhibitor) compared with non-CNI treated. | Mg intake from Food Frequency Questionnaire; serum Mg, urinary Mg | Serum Mg and urinary EGF/creatinine were significantly lower in the CNI treated children with significantly more CNI treated children developing hypomagnesemia and having higher fecal Mg.  Patients who developed hypomagnesemia showed a higher Mg excretion fraction when treated with CNI. The fraction excretion of Mg was 10.4% (1.8%) in the Tx group and 5.9% (1.7%) in the CKD group (p=0.073).  In CNI-treated children who developed hypomagnesemia, the fraction excretion of Mg was increased. The urinary EGF concentration, age, and kidney function are independent predictors of the fraction excretion of Mg. | Study main comparison was Mg intake/ serum and excretion with or without CNI treatment. Difficult to compare with respect to stages of CKD / nephrotic syndrome or Tx. |
| Pontón-Vázquez 2017, Mexico. | Cross sectional | ESKD | 13.6 yr +- 2yr | 55 total  22 PD  33 HD | Comparison of dietary intake, anthropometry and body composition in PD vs HD. | 2 x 24 hour dietary recall  Anthropometric, biochemical (EUC), body composition indicators, mid upper arm circumference, nutrition analysis of lipid, Vit C, sodium intake | Children on PD had significantly greater serum concentrations of biochemical indicators than children on HD. Serum Mg averaged 2.4 (+-0.5mg/dL) for PD vs 1.9 (+- 0.4 mg/dL) for HD.  Serum Mg higher post dialysis if poor growth (z height score <2)  Serum Mg high post PD associated with low Vit A intake. |  |
| Sanjad 2001, Lebanon / Saudi Arabia . | Prospective | Tx | Children; age not reported | 31 | All patients received triple therapy (prednisone, azathioprine, cyclosporine) | Measurement of kidney function, plasma, urine electrolytes and parathyroid hormone (PTH) levels.  Tubular reabsorption of phosphate and urine anion gap were calculated when appropriate. | A significant reduction in serum Mg level was observed in five patients, all receiving Tacrolimus.  Three patients in this group also had proximal RTA. All required supplemental Mg therapy. | Small/modest sample size with retrospective design lending to potential for selection and recall bias |
| Tuokkola 2021, Finland  . | Retrospective observational. | PD/ HD | 0.8 - 8.3 yrs (median = 1.16) | 33 | PD/HD vs recommended intakes / normal reference ranges | Dietary intake (solids + enteral) and serum Mg. | 83% children had a high serum Mg (above reference range). Mg serum levels were overall 1.3 (1.1-15) (Median, IQR)  Serum Mg levels for children on food + feeds were 1.4 (1.2-1.5) (Median, IQR)  Serum Mg levels for children on oral food only were 1.1 (0.9-1.2) (Median, IQR). P = 0.003. (Reference range 0.7-1.0)  Urine output and PD positively associated with lower blood Mg values. | No control groups. Normal reference range may still be suboptimal due to population's lower status than other countries. |
| Zaher 2016, Egypt. | Cross-sectional | HD | 11 ± 3.89 yrs | 25 HDx |  | Serum Mg, | Lower serum Mg levels were associated with vascular calcification in chronic HD children.  Children on HD had significantly lower blood Mg levels than controls. (1.7 ± 0.43 mg/dL vs 2.31 ± 0.12 mg/dL respectively P = 0.001).  Serum Mg negatively correlated with aortic IMT (AIMT). (r=-0.682, P=0.000). AIMT negatively correlated with systolic and diastolic blood pressure (r=0.447, P = 0.025, 0.472, P = 0.017), respectively. |  |

**Table S7. Mg: intervention studies in children**

| **Author, year,, & location** | **Type of study** | **Patients** | **Age** | **N** | **Intervention** | **Outcome measures** | **Findings** | **Potential bias / limitations** |
| --- | --- | --- | --- | --- | --- | --- | --- | --- |
| Chan 1981, USA | Intervention | CRF, HD | 8 (+/-5) | 11 | 1,25 dihydroxyvit D3 (10mg/kg/day increasing up to 35mg/kg/day over 25 days) | Height, height velocity, GFR,  Serum and urinary Mg  Bone age (roentgenogram). | Growth velocity improved in 6 out of 8 children ( 75 %) <12 yrs, and was improved in 1 out of 3 (33% ) children >12 yrs.  The growth velocities of 5 patients increased 122% to 168% over the expected growth velocity for chronologically age and 112% to 168% over the expected growth velocity for bone age.  S-Mg remained normal before and after treatment.  Retention of Mg was observed after treatment for 12 Mo.(38 ± 32 mg / sq m / day); due to increase in intestinal Mg absorption from 29% to 43% (P<0.05).  Treatment did not accelerate rate of deterioration of GFR. | Small study |
| Lewis, 2018 USA | Intervention | Tx | 5-18 yrs | 12 | Mg soy protein vs Mg salts – impact on Mg levels and GI tolerability | Serum Mg, Stool frequency and quality | Mg soy protein is more effective in increasing serum Mg than equivalent mg/kg/d dose of elemental Mg.  Mean serum Mg increased from 1.61 (SD 0.1) on standard Mg oxide to 1.69 (SD 0.1); T(11) = 2.6 , P = 0.02 on Mg oxide bound to soy protein.  In 5 patients who had persisting low serum Mg (<1.7mg/dL) after supplementation with MGP (Mg soy protein) increased dose to 266mg achieved serum Mg > or = 1.7mg/dL (mean 1.75mg/dL, SD 0.06; t (4) = 2.7, P = 0.06).  Stools looser however of same frequency with Mg soy protein. | Use of serum Mg as a clinical marker for Mg status.  Incomplete reporting to gastro-intestinal symptoms |
| Pandango 2023, Indonesia | Double-blind Randomized Placebo-controlled Trial | CKD stage I-IV patients with hyperphosphatemia | 12±4.5 yrs (experimental group)  10.6±4.9 yrs (placebo group) | 31 cases, 29 controls | Mg supplementation 6 mg/kg/d (max 250 mg/d) for 2 mo.  If during follow-up Mg remains > 3.7 mg/dL, the supplementation will be stopped. In case the serum Mg increased but not > 3.7 mg/ dL, Mg supplementation continued using half the dose. | (P-Pi) Plasma Phosphate | P-Pi decreased in both Mg group (5.4 ± 0.9 to 4.8 ± 1.1 mg/dL; p-value = 0.001) and placebo groups (5.1 ± 0.6 to 4.3 ± 1.2 mg/dL; p-value=0.003). eGFR improved in both groups (83.4±25.3 to 118.8±52; p-value=<0.001 and 86.3±28.1 to 96.9 ± 35.8; p-value=0.004); difference in reduction was significantly different (35.4 vs 10.7; p-value=0.045). | Mg concentrations in blood not reported  Adverse effects not reported |

**Table S8. Mg: Non-dietary and other factors for abnormal status**

| **Author, year,, & location** | **Type of study** | **Patients** | **Age** | **N** | **Intervention** | **Outcome measures** | **Findings** | **Potential bias / limitations** |
| --- | --- | --- | --- | --- | --- | --- | --- | --- |
| Chanchlani 2017, Canada | Cohort | Tx | 0 – 18 yrs | 451 |  | Serum Mg | Hypomagnesemia does not confer increased risk hyperglycemia and diabetes. |  |
| Davidovich 2009, Israel | Cross-sectional | CKD | 7-20 yrs | 42 |  | Saliva Mg, serum Mg, dental calculus scores | Dental calculus scores were higher in Kidney patients than healthy controls.  Dialysis patients had salivary Mg (mean = 0.25 mg/dl) significantly lower than in Pre dialysis patients (mean = 0.55mg/dL, P = 0.0004), Tx (mean = 0.57mg/dL, P = 0.002) and control groups (mean 0.43mg/dl, P = 0.017).  Mg values in the Pre-Dialysis group were found to be significantly higher than in the Control group (P = 0.048).  Salivary Mg correlated with dental calculus scores, lower pH and high salivary Ca x P product. | Salivary Mg levels may not indicate body Mg stores |
| de Sequera 2022, Spain | Randomized controlled trial | HD | 63.5 (18.3) and 67.1 (14.1) yrs | 56 | 56 HD patients with bicarbonate three times a week were dialysed for 16 weeks with 3 mmol/L acetate and 16 weeks with 1 mmol/L citrate | Ionic Mg | After 16 weeks of citrate treatment, preHD ionic Ca and Mg were significantly lower and PTH higher than in the acetate period. No differences in the effectiveness of dialysis. Hypotensive episodes were significantly more frequent with acetate than with citrate: 311 (14.1%) vs 238 (10.8%) sessions. The lean mass index increased by 0.96+- 2.3 kg/m2 when patients switched from LD with acetate to citrate. HD with citrate modifies several parameters of bone mineral metabolism. The substitution of acetate for citrate improves hemodynamic stability, producing less hypotension and can improve nutritional status. |  |

**Table S9. Chromium: biochemical status in pediatric studies.**

| **Author, year, & location** | **Type of study** | **Patients** | **Age** | **N** | **Intervention** | **Outcome measures** | **Findings** | **Potential bias / limitations** |
| --- | --- | --- | --- | --- | --- | --- | --- | --- |
| Filler 2017, Canada | Ancillary cross-sectional study to a prospective, longitudinal, randomized controlled trial | CKD + Tx with eGFR < 90 ml/min/1.73m2 (not including dialysis) | 4-18 Yrs | 36 | NA | Serum chromium level | The median Cr level was 0.43 µg/L (0.36, 0.54), which was also significantly greater than the established reference interval; 34 had at least one set of Cr levels above the published reference interval. | Modest sample size.  Water sources not defined. |

**Table S10. Copper: dietary intake in pediatric studies**

| **Author, year,, & location** | **Type of study** | **Patients** | **Age** | **N** | **Intervention** | **Outcome measures** | **Findings** | **Potential bias / limitations** |
| --- | --- | --- | --- | --- | --- | --- | --- | --- |
| Coleman 1992, UK | prospective | CCPD | Peds | 7 | Supplementation of Cu  Receiving  Ketovite tablets (vitamins C, E and B complex) & Cholecalciferol or  Paediatric Kidney Seravit (vitamins A, E, D, C and B complex + trace minerals) | Dietary intake of Cu | All children received nutritional supplements orally or via a gastrostomy button.  Supplements contributed significantly to their nutritional intakes.  Despite Cu intake being below the RDA value, serum copper levels were within the normal range.  No significant difference in serum levels of the micronutrients Paediatric Kidney Seravit vs to Ketovite. | No control group |
| Tamura 1989, USA | cross sectional | PD | 9 Mos to 18 Yrs | 6 | Supplementation withdrawn for 2 mo, then restarted |  | As assessed by a three-day diet diary taken each Mo for four Mos, the authors report a dietary intake of Cu of 39mcg/kg body weight /day (range: 8 to 78mcg), and were lower than recommended intakes. Participants were all prescribed a supplement containing 19.7mcg as part of their usual clinical care. | Old study  Small number of patients |
| Tuokkola 2021, Finland | Retrospective cross-sectional | PD, HD | 0-16 Yrs | 33 | n/a | Estimation of vitamin and mineral intake with a 3-day food record | Median copper intake 156% of RNI (160% on feeds, 142% with food) | No control group |

**Table S11. Copper: biochemical status in pediatric studies**

| **Author, year,, & location** | **Type of study** | **Patients** | **Age** | **N** | **Intervention** | **Outcome measures** | **Findings** | **Potential bias / limitations** |
| --- | --- | --- | --- | --- | --- | --- | --- | --- |
| Coleman 1992, UK | Prospective | CCPD | 6.1 yrs (range 0.3-12.6 yrs) | 7 | Assessed and compared the **serum** levels and **dietary** **intakes** of Cu,  Comparison of nutritional supplements Ketovite and Pediatric kidney seravit | Serum level & dietary intake of Cu | Serum Cu 22.37 +-5.01 (Ketovite group), 22.33 +- 6.22 (pediatric kidney seravit group), normal range 13-24 | Small study |
| Esfahani 2007, Iran. | Evaluation | CKD on HD and conservative treatment + healthy controls | Group 1 11.75 ±3.64 yrs  group 2 10.56  ±3.19 yrs  Group 3 12.02±3.18 yrs | 101 | Evaluated changes in serum Cu levels in two groups of children with CKD – children with CKD who were on regular HD (Group 1, *n*=40) and children with CKD who were on conservative management (Group 2, *n*=31) and in one group of healthy children (Group 3, *n*=30). | Measured serum Cu in three groups of children | The differences in serum levels of Cu among the three groups were not significant.  Serum Cu HD: 0.533 ± 0.231  CKD conservative: 0.62 ± 0.227  Controls 0.61 ± 0.333 |  |
| Esmaeili 2019, Iran | Controlled trial | Children | 5-18 yrs | 200 | Serum levels of Cu, Zn, Se and lead in children with ESKD receiving conservative management, HD or CAPD, and a control group | Serum levels of Cu evaluated using an atomic absorption spectrophotometer and compared between the groups. | There was no significant difference in the serum concentration of Cu among the 4 study groups. | Reference ranges for serum Cu not given |
| Joyce 2020, UK | Retrospective cross-sectional | CKD2-5 | 4-14 | 112 | Review of nutritional blood concentrations measured over a 2 yr period | Nutritional bloods including copper | 87% achieved normal ranges for Cu Deficiencies were seen in 7%,  6% had Cu levels above normal ranges. |  |
| Tamura 1989 | Prospective | PD | 9 Mos to 18 Yrs | 6 | Supplementation withdrawn for 2 mo, then restarted | Serum Cu | Serum Cu within reference range with supplementation and after 2 mo without supplementation | Old study  Small number of patients |
| Youssef 2012, Egypt | Case control | 3 groups:  A: CKD 5 HD,  B: CKD 3-4  C healthy | 5-18 yrs | 50  A:20  B:20  C:10 | Serum Cu | Pre-dialysis venipuncture, fasting state  Atomic Absorption Spectroscopy | Serum Cu was (89.3 *±* 15.1 *μ*g/dL) in HD, (95.6 *±* 16.2 *μ*g/dL) in  CKD 3-4, and (116.6 *±* 13.6 *μ*g/dL) in healthy; significantly higher than in children with CKD (*p <* 0.05). |  |
| Zwolinska 2006, Poland | Controlled trial | PD, HD | 13.2 ±3.5  Group 1  14.8±1.9 Group 2  12.8±3.2  control group | 58 | Evaluate lipid peroxidation (LP) in **plasma** and erythrocytes, erythrocyte antioxidant enzyme activity (superoxide dismutase, SOD; catalase, CAT; glutathione peroxidase, GSH-Px), and concentrations of Cu and Zn as cofactors of SOD and Se as a cofactor of GSH-Px in erythrocytes, **plasma** and in **dialysis** fluid in children with ESKD (group 1 CAPD and group 2 HD) | Evaluation of oxidative stress in children on maintenance dialysis. Examined LP in plasma and red blood cells (RBC), the activities of RBC antioxidant enzymes and their cofactors in erythrocytes, plasma and in dialysis fluid. | Significantly lower concentrations of erythrocyte and plasma Cu as cofactors of SOD and erythrocyte in HD and PD than in controls  Plasma Cu in PD 16.98±1.40, HD 16.25±1.18, in controls 23.45±0.85 μmol/l |  |

**Table S12. Copper: intervention studies and non-dietary and other factors for abnormal status in children**

| **Author, year,, & location** | **Type of study** | **Patients** | **Age** | **N** | **Intervention** | **Outcome measures** | **Findings** | **Potential bias / limitations** |
| --- | --- | --- | --- | --- | --- | --- | --- | --- |
| Chantarogh 2017, Thailand | Case report | CAPD | 5 yr F | **1** | **n/a** | Cu levels  Method NR  Hgb/Hct | Plasma Cu & ceruloplasmin spontaneously reached the normal range within 1 Mo, without supplement  Erythropoietin restarted @ lowither dose (110 units/kg/witheek)  Hgb within the normal limits within 2 Mos.  Anemia in an ESKD due to hypocupremia secondary to hyperzincemia |  |
| Coleman 2002, UK | Interventional | Children | Mean 10.4 | 15 | Children currently prescribed Ketovite tablets were changed to RSM for 3 Mos. **Questionnaires** on palatability, acceptability, and ease of administration were assessed while on Ketovite and after 3 Mos on RSM along with **plasma** levels of Zn, **copper**, folate (**serum**, **red** **cell**), vitamin B(12), and homocysteine. | Plasma levels | Plasma values of copper were within the normal reference range on Ketovite and showed no significant change on RSM. |  |
|  |  |  |  |  |  |  |  |  |

**Table S13. Fluoride: non-dietary and other factors for abnormal status.**

| **Author, year,, & location** | **Type of study** | **Patients** | **Age** | **N** | **Intervention** | **Outcome measures** | **Findings** | **Potential bias / limitations** |
| --- | --- | --- | --- | --- | --- | --- | --- | --- |
| Khandare, 2017  India | Case controlled | Healthy school aged children | 8-15 yrs. | 824 | Assessment of thyroid and kidney function markers in a area with high Fl content in the water. | Serum creatinine, total alkaline phosphatase, parathyroid hormone, 1, 25(OH)2 vitamin D, and osteocalcin | Increased CKD in children in this area compared to controls, decreased vitamin D and abnormal bone-mineral laboratory measures. | May have been influenced by tea consumption; black salt, some variance in Fl levels in the area - all high. |
| Malin 2019  USA | Retrospective | Adolescents in the NHANES cohort with normal kidney function | Adolescents - mean age of 15.4 | 1983 and 1742 in 2 groups | Evaluation of water Fl content compared to biochemical markers - household water samples and serum blood samples were evaluated | Water fluoride content and markers of kidney function | A 1 μmol/L increase in plasma fluoride was associated with a 10.36 mL/min/1.73 m2 lower estimated glomerular filtration rate; 1 mg/L increase in water fluoride was associated with a 0.93 mg/dL lower blood urea nitrogen concentration. | Retrospective study; cross sectional, long-term studies needed; confounders not evaluated. |
| Spak, 1985  Sweden | Prospective Observational. | Children and adolescents with varying stages of kidney function | 5-18 Yrs | 38  12 healthy controls | Assessment of Fl clearance with standard fluoridated drinking water (0.2 mg/L) on children with kidney impairment but varying GFR  *first study to assess fluoride clearance in children | Fl clearance 31.4 +/- 8.8 mL/min in the low GFR group and 45 +/- 9.8 in the group with normal GFR, statistically significant (p <0.1). Higher fluoride plasma concentrations in low GFR group, very low GFR associated with very poor excretion. | Children excrete fluoride less well than adults. Even with mild kidney impairment, children with CKD have further impaired excretion. | Heavily female over male (9 males)  Patients had diseases associated with impaired kidney function but only 5 had GFR lower than normal |
| Warady 1989, USA | Prospective observational | Infants/toddlers on PD | Infants, 0-18 Mo | 4,  27 healthy controls | Measure 3 day food record Fl content, formula content, water content, dialysis fluid - compared to plasma and urine levels | Plasma and urine levels compared to intake | Patients with significantly higher plasma Fl content than controls (p<0.01)  Less Fl excretion with worsening kidney function | Small sample size |
| Xiong, 2007  China | Prospective Observational | Typical children with differing Fl levels in water. | Children – ages unknown. | 210 | 7 groups of children with different levels of Fl content in the water. | serum lactic dehydrogenase (LDH), urine N-acetyl-beta-glucosaminidase (NAG), and urine gamma-glutamyl transpeptidase (gamma-GT) | Those with the highest Fl water content had indications of kidney and liver damage (over 2 mg/L); also highest plasma and urine levels.  Dental fluorosis common with impaired kidney but not liver function. | Potential outside factors such as diet. |

**Table S14. Iodine: dietary intake in pediatric studies**

| **Author, year, & location** | **Type of study** | **Patients** | **Age** | **N** | **Intervention** | **Outcome measures** | **Findings** | **Potential bias / limitations / notes** |
| --- | --- | --- | --- | --- | --- | --- | --- | --- |
| Lindeback 2023, Australia | Cross-sectional | Children with CKD and healthy controls | 2-18 yrs | 36 CKD 82 controls | Nutritional intake and diet quality of children with CKD was determined and compared to control data.  Nutritional intake gathered by Food Frequency questionnaire | Intake of macronutrients, micronutrients and diet quality.  Weight, Height, Body Mass Index | Mean iodine intake was 139 ± 67 µg/day. | Small sample size  Iodine intake not reported in controls  Sample did not include children on dialysis  Sample included children with CKD pre Tx and post Tx.  Children with <80% EER from oral diet excluded. |

**Table S15. Iodine: non-dietary and other factors of abnormal status**

| **Author, year, & location** | **Type of study** | **Patients** | **Age** | **N** | **Intervention** | **Outcome measures** | **Findings** | **Potential bias / limitations / notes** |
| --- | --- | --- | --- | --- | --- | --- | --- | --- |
| Brough 2006,  UK | Case report | CCPD  iodine exposure from povidone-iodine cap in PD catheter | infants | 2 |  | Plasma iodine concentration  PD-fluid iodine concentration  urine iodine concentration | Concentration of iodine in the PD fluid was higher than that in the plasma  Iodine concentrations in PD (PD) fluid were found to be higher at the end of the first cycle (11.4 mol/l) than at the end of the twelfth cycle (1.55 mol/l). | Iodine in povidone-iodine 10% (Betadine), which is in the cap used at the end of the PD catheter may have been the cause for iodine exposure |
| Cohen 2019,  France | Case report | CKD, iodine injection in cystography | newborns | 2 |  | Thyroid function tests T4, TSH  urine and plasma iodine concentrations,  clinical signs | Hypothyroidism  iodine plasma level 2.41 & 57.8 μmol/l (normal 0.28– 0.8) and iodine urine excretion 4.03 & 116.4 μmol/l (normal 0.35–1.6) with iodine/urinary creatinine ratio 3.88 & 103.95 μmol/mmol (normal 0.03–0.3).  Abnormal hypotonia and hypoactivity | Intra vesical iodine injection during a cystography as a source of iodine |
| Eloot 2010,  Belgium | Case report | Burns patients with elevated iodine levels |  | 2 | continuous HD |  | In patients with elevated iodine levels, especially when associated with kidney failure, HD with a minimum 12-h duration with sufficient blood flow should be the first choice to remove iodine. |  |
| Kutlay 2015,  Turkey | Case-control | HD  iodine-deficient community | HD 42.94 +/- 11.88, controls 40.20 +/- 10.72 | 87 HD, 169 controls |  | Thyroid gland using ultrasonography  TSH, T3, T4 | Nodular goiter in 32.2% in HD and 23.5% in controls  Hypothyroidism in 3.4% HD, 0.6% controls | Iodine blood / urine concentrations not reported |
| Lebkowithska 2003, Poland | Case.control | HD, CAPD, RTx | HD 56 +- 18, CAPD 51+-11, RTx 44+-12 | 48 RTx, 32 HD, 26 CAPD, 40 controls |  | Thyroid gland, TSH, T3, T4, urinary iodide | Prevalence of goiter was 100% in Tx, 50% in HD, and 25% in CAPD patients |  |
| Mannemudhu 2020, USA | Case report | PD  povidone-iodine cap | 2 witheeks - 17 mo | 4 |  | Serum iodine concentration | Hypothyroidism | Source of iodine exposure in each child was a povidone-iodine-impregnated gauze in the sterile transfer set cap associated with PD |
| Pakfetrat 2017,  Iran | Cross-sectional | HD + controls | cases 57.2 ± 17.2 yrs, controls 56.6 ± 16.8 yrs | 86 + 86 |  | Physical examination / goiter  ultrasonography  T3, T4, TSH  Urinary iodine excretion | 29% of patients had goiter  HD patients had a higher frequency of reduced FT3 (40.9% vs. 4.6%, P < 0.01) and increased TSH (18.6% vs. 8.1%, P < 0.03  Urinary iodine excretion 4.6+-1.4 in HD patients, 17.6+-9.8 in controls, p = 0.035 |  |
| Rasche 2018,  Germany | Prospective cross-sectional | HD, HDF | 66 +- 15 yrs | 170 | No iodine containing medication  No iodine containing contrast media for 1 Mo prior laboratory tests  2 centers | TSH  serum iodine  (+ prolactine,albumin, T4, T3) | Median iodine serum concentrations differed between centers (0.55 (0.3 to 26.5) μmol/l vs. 0.50 (0.3 to 9.6), p = 0.001  Serum iodine correlated with fT4 (p<0.001, r=0.43) and albumin (p=0.001, r=0.245) but not with morphological signs. | Higher albumin, a marker for a healthier HD patient, was  associated with higher fT3, fT4, use of HF membranes, residual excretion rate, and dialysis time per week.. We conclude that not only unchangeable risk factors will contribute to the normalization of  the pituitary-thyroid axis, also use of modern HD technologies and time on dialysis per week might influence the pituitary-thyroid axis  positively. |
| Ramirez 1976, USA | cross-sectional | HD, CRF, controls | 14-70 yrs | 29 HD, 40 CKD, 40 controls |  | Serum iodide  thyroid gland responsiveness by thyrotropin administration. | T3 below normal in HD and CRF patients  serum iodide concentrations elevated: 1.0+-.05 in controls, 3.9+-3.2 in CEF, 3.4+-1.9 in HD.  No correlation between the level of serum iodide and the degree of kidney failure. | Old study - HD procedure different from today? |
| Sanai 2008, Japan | cross-sectional | HD, hypothyroid | 69.1 +/- 8.8 Yrs | 141 HD; intervention in 14 with hypothyroidism | Iodine restriction in diet (avoidance of seaweed and tangles) | TSH | Goiter in 19 (13.5%). Primary hypothyroidism in 14 (9.9%). After iodide restriction, the serum TSH level decreased in all the patients from a mean of 16.49 A+/- 22.80 to 4.44 A+/- 3.35 mU/l after 1 Mo, 4.25 A+/- 2.24 mU/l after 2 Mos and 3.97 A+/- 2.22 mU/l after 3 Mos. ; no effect on T3 and T4 | Habitual high iodine intake  Serum iodine concentrations not reported |
| Sato 1992, Japan |  | mild to severe CKD | adults | 245 primary hypothyroidism, of which 36 with CKD | Iodine restriction in diet | TSH, T3, T4  serum and urinary iodine, iodine loading test, perchlorate discharge test. | Elevated serum non-hormonal iodine level (median 236, range 67-15,591 micrograms/l  Recovery of thyroid function in 30 (83%) of patients after iodine restriction in diet  In iodine loading test, elevation of serum iodine greater and urinary iodine less in patients with CKD vs controls |  |
| Selvathesan 2023, new Zealand | case-report | PD | infant | 1 |  | Serum iodine  dialysate iodine. | Iodine toxicity leading to AKI  Source of iodine PD-catheter cap |  |
| Takeda1993,  Japan | cross-sectional | HD, CAPD | 52.4+-13.8 | 93 HD; intervention in 3 with hypothyroidism | Iodine restriction in diet | TSH, serum iodine, iodine percholate discharge test | 3 patients (3.2%) with hypothyroidism, THS above 40 µU/ml had an enlarged thyroid gland; , no history of thyroid disease or medication affecting thyroid function.  Habitually eaten iodine-rich foods  Showed an enlarged thyroid gland  elevated TSH, Iodine; iodine restriction TSH decreased from 44.6 / 90.6 / 43.2 -> 3.6 / 3.2 / 9.4 µU/ml, decrease in serum Iodine | Habitual iodine intake high in area, no underlying thyroid disease |
| Vulsma 1991, Netherland | case report | PD | 3 yrs and 18 Mos | 2 |  | Plasma T5, thyroid-stimulating hormone, thyroglobulin, radio-iodine uptake | PD catheter cap as a source of iodine toxicity |  |

**Table S16. Manganese: biochemical status in pediatric studies**

| **Author, year, & location** | **Type of study** | **Patients** | **Age** | **N** | **Intervention** | **Outcome measures** | **Findings** | **Potential bias / limitations** |
| --- | --- | --- | --- | --- | --- | --- | --- | --- |
| Joyce 2018, UK | observational | PD  HD | 2.8-14.4 yrs | 47,  PD 19  HD 28 | Different vitamin and mineral supplementation | Serum Mn | 60% within normal range and  40% above normal ranges for Mn.  Total 234 (111), HD, 255 (127) PD 202 (74) nmol/L (normal range 73–210 nmol/L)  23/47 had oral nutritional supplements or enteral feeds during the observation. | No dietary intake data  Variability to adherence to micronutrient supplementation and variability in HD membranes used |
| Verma 2023,  Canada | Cross-sectional  Prospective Longitudinal  RCT | CKD stage 1-4  Pre-dialysis | 0-19 Yrs | 42 | Longitudinal RCT on Zn supplementation | Plasma Mn | Median Mn plasma levels were normal  Trend towards lower values with lower eGFR  14% had low plasma Mn levels overall  Mn z-scores significantly lower than and decreased compared to reference general population data | Bias towards milder CKD, no baseline assessment on variation of diet, supplement intake or local water content.  Reference data not controls |

**Table S17. Selenium: dietary intake in pediatric studies**

| **Author, year, & location** | **Type of study** | **Patients** | **Age** | **N** | **Intervention** | **Outcome measures** | **Findings** | **Potential bias / limitations** |
| --- | --- | --- | --- | --- | --- | --- | --- | --- |
| Lindeback 2023, Australia | Cross-sectional | Children with CKD and healthy controls | 2-18 yrs | 36 children with CKD and 82 healthy controls | Nutritional intake and diet quality of children with CKD was determined and compared to control data.  Nutritional intake gathered by Food Frequency questionnaire | Intake and diet quality. | Average intake = 87 (+/036) ug/d, which is above RDA. | No control group data for Selenium assessment. Did not express as % of requirements, despite recommended intake varying depending upon age.  Small sample size,  Children with <80% EER from oral diet excluded. |
| Tuokkola 2021, Finland | Retrospective | Children (dialysis patients - mix of PD and HD) | 0-16 yrs | 33 | Review patient records to estimate dietary intake  3-day food records | Dietary and total intake | The median dietary intake was below age-specific  recommendations (median = 85% of requirements (70 to 101%), but met these recommendations once supplementations were included (median = 126% (88 to 186)). | No control group. Normal reference range may still be suboptimal due to the population's lower status than other countries.  Small sample size.  Does not separate PD and HD. |

**Table S18. Selenium: biochemical status in pediatric studies**

| **Author, year, & location** | **Type of study** | **Patients** | **Age** | **N** | **Intervention** | **Outcome measures** | **Findings** | **Potential bias / limitations** |
| --- | --- | --- | --- | --- | --- | --- | --- | --- |
| Esmaeili 2019, Iran | Prospective observational. | Children Pre-D CKD (mean eGFR=8), PD, HD, controls. | Mean = 12.7 Yrs. | Pre-D CKD = 14, PD=45, HD=63, controls=78. | CKD vs controls. | Serum Se concentrations. | Serum Se lower in dialysis pts, but not so in Pre-D CKD. Lowest Se was in the PD group (100.44 ug/L), but this is in normal reference range. Therefore, this may not be clinically important. | No dietary intake. |
| Joyce 2018, UK | Retrospective observational. | Children on PD / HD | Median = 11.4 yrs (2.8, 14.4) | 47 | PD/HD vs normal reference ranges | Serum Se concentrations. | Serum Se total 0.97 (SD 0.59), HD 0.94 (0.66), PD 1.02 (0.46)  4% (3% of PD, 7% of HD) were below the hospital's normal reference range of 0.44–1.43 μmol/L. 7% (3% and 13% for PD, and HD) were above the reference range. | No control groups. Normal reference range may still be suboptimal due to the population's lower status than other countries. No dietary intake. |
| Joyce 2020, UK | Retrospective observational. | Children with pre-D CKD, stage 3 to 5. eGFR median = 28 (21, 37). | Median = 8.97 (4.24, 13.80) Yrs. | 112 total 104 with Se concentrations. | CKD vs normal reference range. | Serum Se concentrations. | Median serum Se = 1.17 (1.02, 1.33) umol/L. 1% below, and 14% above normal reference range (0.44–1.43 μmol/L). No difference between diet and tube fed/Oral Nutrition Support groups. | No control groups. Normal reference range may still be suboptimal due to the population's lower status than other countries. No dietary intake. |
| Ortac 2006, Turkey | Prospective observational. | Children, PD, HD, controls. | Mean 11.1 yrs (4.4) | 32 Pre-D CKD, 42 PD, 19 HD, 34 controls. | CKD vs controls. | Hair Se concentrations | Hair Se and GPx activity lower in CKD vs. controls. No correlation between eGFR and Se / GPx. | Hair Se status is research practice. No description of selection of control group. Controls had a mean eGFR = 84 (not normal). |
| Tuokkola 2021, Finland | Retrospective cross-sectional | Children on PD / HD | Median = 1.16 (0.8-8.3) Yrs. | 33 (32 with plasma concentrations). 29 with dietary intakes. | PD/HD vs recommended intakes / normal reference ranges | Serum Se concentrations. | Median serum Se 1.1 (0.9-1.3) umol/L. Normal reference range = ,1 y: 0.42-0.90, 1-4 y: 0.41-1.07, 5-9 y: 0.52-0.94, 10-16 y: 0.51-1.04. 3.1% below, and 68.8% above this normal reference range. No difference between diet and feed subgroups for plasma Se, but diet only subgroup had lower intake. None of those with a low Se intake had a low plasma Se. Intake correlated with plasma Se. | No control groups.  Normal reference range may still be  suboptimal due to the population's  lower status than other countries. |
| Zwolinska 2004, Poland | Prospective observational. | Children Pre-D CKD, HD, controls. | Mean 12 yrs | 46 Pre-D CKD, 21 HD, 27 controls | CKD vs controls. | Plasma and Erythrocyte concentrations and markers of oxidative stress. | Plasma and Erythrocyte Se lower than healthy controls, although in the normal reference range. | Dietary intake not examined. Other findings (vitamin A) also lower than healthy controls which is not in agreement with other studies. |

**Table S19. Zn: dietary intake in pediatric studies.**

| **Author, year, & location** | **Type of study** | **Patients** | **Age** | **N** | **Intervention** | **Outcome measures** | **Findings** | **Potential bias / limitations / notes** |
| --- | --- | --- | --- | --- | --- | --- | --- | --- |
| Coleman 1992, UK | prospective | CCPD | 6.1 yrs (range 0.3-12.6 yrs) | 7 | Ketovite tablets (vitamins C, E and B complex) & Cholecalciferol  or  Paediatric Kidney Seravit (vitamins A, E, D, C and B complex + trace minerals). | Dietary intake of Zn. , | All children received nutritional supplements orally or via a gastrostomy button  Supplements contributed significantly to their nutritional intake of Zn. | Small study |
| Foreman1996, USA | Prospective observational | CKD | 18 Mos - 10 Yrs | 120 | n/a | 4-day food records  Serum Creatinine,  Anthropometry x 6 mos | Zn intake at the lower end of RDA |  |
| Lindeback 2023, Australia | Cross sectional | 36 CKD  82 Controls | 2-18 yrs inclusion  (12.3 +/- 4 yrs) | 118 total | Food Frequency Questionnaire. | Macro and micronutrient intakes | Zn intake (mg/d): 12 +/- 149 CKD vs. 14.1 +/- 5.7 controls (*p =*  0.04)  %RDI 202 +/- 149 CKD vs 262 +/- 106 controls (*p =* 0003) | Use of Food Frequency Questionnaire. |
| Tamura 1989, USA | Prospective Observational | CAPD for an average of 12 Mos (range 5-27 Mos) before beginning | 9 mos – 18 yrs | 6 (3M, 3F) | During the present study, the oral supplementation of trace minerals was discontinued for 2 consecutive Mos in the middle of the 4-Mo study period | Nutritional status of Zn  dietary intakes of Zn were estimated for each patient based on 3-day dietary diaries. | Zn intake lower than RDA | Old paper, small patient number |
| Tuokkola 2021, Finland | Retrospective cross-sectional | PD, HD | 0-16 yrs | 33 | n/a | Dietary intake was estimated from feed prescriptions and 3-day food records.  Including vitamin/mineral supplementation;  Vitamin and mineral determinations were performed as part of routine care  Fasted | Food records or adherence to dietary prescription of feeds were available for 29 children.  Children not on feeds had a low intake of Zn, Insufficient intake was corrected with supplementation.  No children with/ below normal concentrations of Zn but lower than children with normal food vs feeds. P< 0.05  Median intake from food 6.1 (5.1-7.2) mg 116 (86-135)% of RDA mg, total intake 10.4 (6.7-14.0) mg, 156 (129-254)% of RDA | No control groups |

**Table S20. Zn: biochemical status in pediatric studies.**

| **Author, year, & location** | **Type of study** | **Patients** | **Age** | **N** | **Intervention** | **Outcome measures** | **Findings** | **Potential bias / limitations / notes** |
| --- | --- | --- | --- | --- | --- | --- | --- | --- |
| Casey 1981, USA | Case control | Dialysis 1-6 Mos    HD (9)  PD (2)  Healthy children (8) (4-15 yrs)    None on Zn supplement | Children 6-19 yrs | 19 | n/a | Blood Pre/post dialysis session.  Blood in acid-washed tubes containing Zn-free heparin.  Hair samples from the occipital region of the head, cut as close to the scalp as possible.  Zn & Cu emission spectrophotometry.  Growth/anthropometry. | Serum Zn increased post dialysis but not significant  Serum Zn not diff from control group  Serum Zn repeated in 7 patients post 3 mos (80 +/- 29.9mcg/dl), & in 5 patients post 1 yr (101.6 +/- 30.6mcg/dl).  Mean hair Zn level in seven of the patients was 166 +/- 44.5mcg/gm.  All individual values were within the normal range (> 105/~g/gm) laboratory. | Old paper |
| Coleman 2002, UK | Observational. | CKD with/ GFR <25 (N= 11) or CPD (N=2) & HD (N=2) | Children    Age: 1.1-16 yrs | 15 (10 male) | Serum values    3 Mo supplementation Ketovite changed to RSM  Kidney Micronutrient Supplement (vitamins C, E, K, and B complex, copper, Zn) vs “Ketovite” (vitamins C, E, K, and B complex) | Fasting blood samples standard kidney function tests, plasma & red cell folate, vitamin B12, Zn, copper, & homocysteine levels  H high-performance liquid chromatography with fluorescence detection | In CCPD patients  On Ketovite 13.66+-1.94  On Pediatric Renal Seravit 22.54+-15.84  No significant difference  Reference range 11-20 µmol/L | 2 fed via GB overnight |
| El Tayeb 2009, Egy | Cross sectional case control | HD | Children 9-17 Yrs | 24 (15 male, 9 female)    15 healthy age matched controls | Serum values | Zn, Mg, PTH, Ca, PO, Alkaline Phosphatase, urea, & creatinine  Pre HD. atomic absorption/flame emission spectrophotometer Fasted state NR. | Zn/ levels significantly lower vs controls.  negative correlation between PTH & Zn.  Children with CKD on dialysis had significantly lower serum Zn than healthy controls.  Mean serum Zn in children with CKD on dialysis was 14.56 Umol/L ± 6.09.  Mean serum Zn in healthy controls was 19.70 Umol/L ± 5.52. (P <0.05)  There was a significant negative correlation between serum Zn levels and PTH in children with CKD on HD in the patient group ( r= -0.41, p<0.05). |  |
| Esfahani 2007, Iran | Case control, cross sectional | HD    Group1: HD, Group2: CKD non-dialysis  Group3: healthy controls    Group 1 with 2 subgroups - <18 Mos (A) & >18 Mos (B) | Group 1 11.75 ±3.64 yrs  group 2 10.56  ±3.19 yrs  Group 3 12.02±3.18 yrs | 101 | Serum values    Impact of dialysis duration | 6-10 hours of fasting. disposable plastic syringes; washed with acid to remove any contamination.  Zn, Cu, Se, PIXE  Other nutrients: Cobalt, Mn, Cr, Ni | Mean serum levels Zn, lower group 1 vs Group 2 & 3  not significant Zn, ~~i~~ Group 2 & 3  Zn, significantly lower subgroup B vs A  Inverse linear relation between period of HD & serum levels of Zn, | Nutritional state not analyzed, or diet assessed. Patients had similar geography, diet, nutritional habits. No limits on food provision. |
| Esmaeili 2019, Iran | Cross sectional Case control, observational | ESKD non- dialysis, or HD or CAPD  4 groups:  HD,  PD, non-dialysis, control | Children      5-18 yrs | 200 | Serum values | Fasting state NR.  atomic absorption spectrophotometer | No significant difference in the serum concentrations of Zn, between healthy children and non-dialysis group, HD, or PD  Zn levels significantly lower in HD & PD vs healthy children or in non-dialysis groups | Intakes similar, unrestricted |
| Joyce 2018, UK | Retrospective | PD (19), HD (28) | Children (11.4 (2.8, 14.4)) yrs | 47 (24 female) | n/a | Nutritional blood concentrations  average values of vitamin and trace element measurements first 12 Mos (q 3 mos) following commencement of dialysis during the study period were calculated for each patient  Cu, Zn, folate and vitamins A, D, B12 and E.  Assay NR,  Assessed enteral feed & kidney vitamin compositions | 37% Zn within normal range;  Deficiencies in 43%,  above normal in 20% children |  |
| Joyce 2020, UK | Retrospective cross sectional | CKD 3-5, non dialysis | Children  median (IQ1, IQ3) age 8.97 yrs (4.24, 13.80) | 112 (70 M) | 2-year longitudinal analysis;  Performed yearly | Serum Zn assessment & diet intake;  Fasted state NR. | Zn: 60% normal ranges for serum levels (11.6 (10.4, 12.8 umol/L)); Deficiencies: 35% for Zn; Above range: 5% Zn  Reference range: 11-19 umol/L. |  |
| Tamura 1989, USA | Prospective | CAPD    CAPD for an average of 12 Mos (range 5-27 Mos) before beginning | 9 mos – 18 yrs | 6 (3M, 3F) | During the present study, the oral supplementation of trace minerals was discontinued for 2 consecutive Mos in the middle of the 4-Mo study period | Zinc studied during a single dialysis exchange & over a 3-day period.  Blood samples at the end of a usual overnight CAPD exchange, and also after a usual 4-hour CAPD dwell.  Dialysate for 3 consecutive days.  Dialysates from each 24-h period were combined, volume measured, and a portion was used to determine Zn and calculate daily losses of these minerals into the dialysate. Zn levels of the fresh dialysate of the same lot number were determined for comparison in each patient.  Zn concentrations in serum and dialysis solutions were measured by flame atomic absorption spectrophotometric method | Serum Zn levels declined without supplement.  1 Mo after oral supplements had been restarted, serum Zn levels returned to normal.  Zn in dialysis exchange indicated that the patients absorbed Zn. | Old paper, small patient number |
| Tuokkola 2021, Finland | Retrospective cross-sectional | PD, HD | 0-16 yrs | 33 | n/a | Serum Zn | No children w/ below normal concentrations of Zn. Lower levels in children eating normal food than feeds (P< 0.05.9 All children eating normal food were using supplements. | No control group |
| Wihelm 1986, Germany | Observational | 16 healthy  (7-17 yrs)    6 Tx (10-20 yrs) (3 yrs post Tx)    6 HD (12-16 yrs) | Children | 28 | Dialysate, urine, serum | Freshly prepared dialysis fluid, urine samples.  All glass and plastic labware was decontaminated.  No labware or reagents were found to contain detectable Zn.  Zn measured via flame atomic absorption spectroscopy.  Pre & Post HD samples. | No Zn deficiency.  Serum Zn concentrations increased via result of high metal levels in dialysis fluid;  Dialysis increased serum Zn (P < 0.05) concentrations  After kidney Tx, no alterations in  Zn concentrations vs healthy children except whole blood Zn urinary excretion decreased in the following order: healthy, kidney Tx and dialysis children.    Post-dialytic serum concentrations of Zn (P < 0.01) | Old study |
| Youssef 2012, Egypt | Case control | 3 groups:  A: CKD 5 HD,  B: CKD 3-4  C: healthy | Children    5-18 yrs | 50  A:20  B:20  C:10 | Serum values | Pre-dialysis venipuncture, fasting state  Atomic Absorption Spectroscopy | Serum Zn (74.4 ± 12.6 mcg/dL) in HD, (76.7 Â± 13.5 mcg/dL) in CKD3-4; no significant difference between HD and CKD3-4  HD & CKD3-4 vs controls  Controls (97.7 ± 14.3 mcg g/dL) (p< 0.05).  *correlation between Zn, Cu with other parameters either (demographic or laboratory), significant negative correlation with urea, creatinine & a positive correlation with serum iron level* |  |
| Zwołińska 2006, Poland | Case control | Group 1: CAPD (10)  Group 2: HD(21) | 13.2 ±3.5  Group 1  14.8±1.9 Group 2  12.8±3.2  control group | 31 | n/a | Plasma overnight fasting by the healthy subjects & children on CAPD.  HD: pre & 20 mins into session.  Dialysis fluid in both groups.  SOD (superoxide dismutase) activity was assayed according to Misra and Fridovich based on the ability of SOD to inhibit autoxidation of epinephrine at alkaline pH (pH 10.2), & expressed in U/g Hb.  Plasma & erythrocyte Zn via atomic absorption spectrophotometry. | Plasma levels PD 12.23±0.70, HD 11.83±0.95, controls 14.58±0.52  ESKD lower Se, in plasma & erythrocytes vs healthy subjects  activity of SOD, GSH-Px, CAT, concentrations of erythrocyte and plasma, Zn were lower in children with ESKD vs in controls  increased oxidative stress occurs in children on maintenance dialysis, independent of dialysis modality.  Oxidative stress is aggravated during every single HD session in children.  The activity of the enzymatic antioxidant defense system is reduced in red blood cells of pediatric dialysis patients. |  |

]

**Table S21. Zn: non-dietary and other factors of abnormal status**

| **Author, year, & location** | **Type of study** | **Patients** | **Age** | **N** | **Intervention** | **Outcome measures** | **Findings** | **Potential bias / limitations / notes** |
| --- | --- | --- | --- | --- | --- | --- | --- | --- |
| Chantarogh 2017, Thailand | Case report | CAPD | 5 yr (F) | 1 | n/a | Zn levels  Cu levels  Method NR  Hgb/Hct | Initially Hyper Zn; Plasma Zn level gradually decreased from 224 to 112 µg/dl (normal range 70–120 µg/dl) after Zn supplementation was discontinued  Plasma Cu & ceruloplasmin spontaneously reached the normal range within 1 Mo, without supplement  Erythropoietin restarted @ lower dose (110 units/kg/week)  Hgb within the normal limits within 2 Mos.  anemia in an ESKD due to hypocupremia secondary to hyperzincemia |  |

**Table S22. Zn: intervention studies in children.**

| Eggert JV, 1982  USA | double blind crossover | CKD | 10 yrs (range 0.5-19 yrs) | 14 | Zn supplementation x 6 mos | RBC Zn  Taste Acuity  Energy intake | Zn supplementation increased red blood cell Zn concentrations & taste acuity  In those with less advanced kidney failure (serum creatinine < 5.0 mg/dl)  also improved caloric intake  No changes in growth velocity |  |
| --- | --- | --- | --- | --- | --- | --- | --- | --- |
| El-Shazly 2015, Egypt | Prospective RCT | HD | Peds  Group I: (21 females and 19 males) was 13.8±3.1 Yrs.  Group II: ten males and ten females) was 12.5±3.1 Yrs. | 60  Group I: Zn supplement (40)    Group II: placebo (20) | Group I: Zn sulfate (50–100 mg Zn sulfate [equivalent to 11–22 mg elemental Zn]  Group II: cornstarch  BID dose x 90 days | BMI  Serum Zn (pre HD, fasted)  Zinc-assay kit it is a direct colorimetric assay based on the 5-Br-3′-phosphoadenosine-5′-phosphosulfate (5-Br-PAPS) method without deproteinization; 0 & 90 days  Leptin (pre HD, fasted); 0 & 90 days  DRG leptin enzyme-linked immunosorbent assay  Anthropometry  Nutritional history (regarding quality and quantity of food rich in Zn) | Zn supplementation = significant increase in mean serum Zn level & BMI  Serum leptin decreased significantly post supplementation in children under HD.  significant negative correlation was observed between serum Zn & leptin levels as a result of Zn supplementation |  |
| Escobedo-Monge MF 2019, Peru | RTC, (1:1), multicenter | CKD (23 F, 25M)  (HD, CAPD, CKD) | <18  1-18 yrs | 48  (32, 5, 10) | 15 vs 30 mg dose  Phase I: nutritional status 3 Mos  Washout  Phase II: x 12 mos | Serum Zn, anthropometrics, CRP, Alb  Fasting blood samples at the start & the end of the experimental study were collected (before dialysis in patients in HD) | No difference in Zn, CRP, Alb.  Positive association with Zn level and Alb pre & post.  In both groups of ZS, small but  positive & significant change in body mass & normalization in BMI Z-score,  hypoalbuminemia, hypozincemia & high CRP, especially with 30 mg/day of ZS. | No control group, no assessment of dietary intake. |
|  |  |  |  |  |  |  |  |  |

Supplementary material S23. Research recommendations for the assessment and management of magnesium and trace element status in children with CKD.

There is a dearth of data specific to the topic of Mg and TEs in the pediatric CKD population. As noted, we propose that the most pressing topic is to identify and characterize the Mg and TE profile associated with changing kidney function (e.g., how blood concentrations of Mg and TEs change with declining GFR). Furthermore, this requires characterization in the context of pertinent CKD-associated sequelae directly influencing Mg and TE status such as proteinuria, anuria, and polyuria.

We have the following specific recommendations for future research in pediatric CKD2-5D/T:

- 1) derive data-driven recommendations regarding dietary requirements for Mg and TEs
- 2) ascertain clinically safe ranges for Mg and TEs biochemical status
- 3) define clinical scenarios when blood-based Mg and TEs surveillance should be performed
- 4) establish the relationship between the Mg and TE profile with multi-system CKD-associated comorbidities such as cardiovascular disease, poor growth, neurodevelopmental delay, thyroid dysfunction, and anemia
- 5) develop and validate functional assays to determine TE status
- 6) assess prospectively dietary and medical interventions in cases of Mg and TE deficiency and excess
- 7) develop and test age- and disease-appropriate TE supplements
